# Alternated activation with relaxation of periosteum stimulates bone modeling and remodeling

**DOI:** 10.1038/s41598-024-61902-w

**Published:** 2024-05-15

**Authors:** Nikola Saulacic, Hiroki Katagiri, Masako Fujioka-Kobayashi, Serge L. Ferrari, Maude C. Gerbaix

**Affiliations:** 1 Department of Cranio-Maxillofacial Surgery, Faculty of Medicine, University of Bern, Bern, Switzerland; 2 Advanced Research Center, The Nippon Dental University School of Life Dentistry at Niigata, Niigata, Japan; 3 Department of Oral and Maxillofacial Surgery, The Nippon Dental University School of Life Dentistry at Tokyo, Tokyo, Japan; 4 Service of Bone Diseases, Department Medicine, Faculty of Medicine, Geneva University Hospital, Geneva, Switzerland

**Keywords:** Bone, Periosteum, Distraction osteogenesis, Bone modeling, Bone remodeling, Bone, Preclinical research

## Abstract

Gradual elevation of the periosteum from the original bone surface, based on the principle of distraction osteogenesis, induces endogenous hard and soft tissue formation. This study aimed to assess the impact of alternating protocols of activation with relaxation (periosteal pumping) on bone modeling and remodeling. One hundred and sixty-two adult male Wistar rats were used in this study. Four test groups with different pumping protocols were created based on the relaxation applied. Two control groups underwent an activation period without relaxation or only a single activation. One group was sham-operated. Periosteal pumping without period of activation induced gene expression in bone and bone remodeling, and following activation period enhanced bone modeling. Four test groups and control group with activation period equaled the values of bone modeling at the end-consolidation period, showing significant downregulation of *Sost* in the bone and periosteum compared to that in the sham group (*p* < 0.001 and *p* < 0.001, respectively). When all test groups were pooled together, plate elevation from the bony surface increased bone remodeling on day 45 of the observation period (*p* = 0.003). Furthermore, bone modeling was significantly affected by plate elevation on days 17 and 45 (*p* = 0.047 and *p* = 0.005, respectively) and by pumping protocol on day 31 (*p* = 0.042). Periosteal pumping was beneficial for increasing bone repair when the periosteum remained in contact with the underlaying bony surface during the manipulation period. Following periosteal elevation, periosteal pumping accelerated bone formation from the bony surface by the modeling process.

## Introduction

The periosteum is a natural scaffold and a source of cells and bioactive factors that promote osteogenesis^1^. Activation, expansion, and differentiation of periosteal stem/progenitor cells are essential in building a template for subsequent neovascularization, bone modeling, and remodeling^2^. Modeling-based bone formation (MBBF) is controlled by genetic and environmental factors, such as physical strain and hormonal factors. After adolescence, MBBF is restricted to specific stimuli, such as loading^3^. Throughout life, remodeling-based bone formation (RBBF) significantly contributes to bone formation. RBBF occurs on resorption lacunae; it is less prominent on cortical than on cancellous bone surfaces but also occurs inside the compact cortical bone characterized by a cutting cone^4–7^.

Mechanical loading has been specifically considered and targeted in therapies as a naturally attractive route for accelerating bone gain^8^. Loading induces adaptive changes in bone structure and geometry owing to altered bone resorption/formation activity. Changes in the local environment experienced by periosteal cells during mechanical loading contribute to their expansion and are correlated with the greatest areas of new bone apposition^9^. Compressive strain is imposed on osteocytes through the compression and relaxation of bone extracellular matrix, which is caused by cyclic physiological loading and unloading. Contact with the underlying bone is fundamental for the periosteal cells to express their osteogenic capacity^10,11^. Nevertheless, periosteal stripping from the bony surface performed prior to the bone grafting disturbs this environment and negatively contributes to the osteogenic process^10,12^. The viability of the elevated periosteum is heavily impaired, leading to delayed healing and several weeks of recovery^12^. Thus, the new bone must originate only from the endosteum and marrow spaces of the pristine bone.

Periosteum is the most crucial structure for successful bone regeneration during fracture healing and distraction osteogenesis (DO). The principle of DO was based on a progressive elongation of the bone fragments created through osteotomy, resulting in an endogenous hard and soft tissues formation^13^. The local mechanical environment influences the regenerative pathways during DO. Bone formation during DO is clonally derived from skeletal stam cells after reverting to a developmental neural crest-like state^14^. Tevlin et al**.**^15^ further revealed that a predominance of innervated skeletal stam cells are dominated by pathways related to bone formation. However, it remains unclear whether intrinsic osteogenic growth factors induced by distraction forces function at their maximum potential. Osteoblasts exposed to different stresses may activate distinct pathways, resulting in proliferation and differentiation profiles unique to the type of stress applied^16,17^. Cyclic mechanical stretch (5 s stretch/5 s relaxation) stimulates osteoblasts at the early and late stages of differentiation at a moderate level (neither too high nor too low)^18^. Active dynamization or the so-called "accordion maneuver" was used in long bones to stimulate distraction regeneration and reduce the overall treatment period^19,20^ or enhance the bony union at the docking site^21^. The alternation protocol was successfully performed in long bone distraction during^22,23^ or at the end of the activation period^23^; however, a standard protocol has not been established^24^.

The principle of DO can be also applied to maintain the osteogenic capacity of elevated periosteum. During periosteal distraction osteogenesis (PDO), the periosteum is gradually distracted from the bone surface by a perforated plate or mesh^25^. Compared to conventional DO, the distraction gap formed by PDO is bordered by the original surface of the bone base and by the periosteal (i.e., cambial) layer. The simultaneous formation of hard and soft tissues during PDO is a unique method of endogenous tissue engineering. The procedure is less invasive and more refined than bone grafting as it avoids harvesting of the bone block grafts and prevents potential inflammation related to the use of biomaterials. The histological features of new bone apposition associated with PDO in different animal models, anatomical sites, and devices have been well described^26^. The differences between findings may be due to an increase in the production of bone scaffold and mineralization. The molecular mechanisms and the implicated pathways that control the differentiation program of mesenchymal stem cells during PDO remain poorly understood. The periosteum might be osteogenic, or act as a sleeve that mechanically activates the underlying granulation tissues, i.e. indirectly regulate cell differentiation and proliferation from the cortical bone. How PDO affects the remodeling of the pristine bone is still unknown.

Our preliminary data indicated that the distraction protocol alternated with relaxation (periosteal pumping [PP]) enhanced the apposition of new bone on the calvaria of rats^27^. Furthermore, introducing relaxation after two activations was beneficial for regenerating extended bone defects compared to the standardized PDO protocol^28^. Hence, the nature and kinetics of bone formation may be influenced by varying relaxation moments. The present study aimed to examine the relationship between different modes of periosteal manipulation and endogenous bone formation at different time points in a rat calvarial model. The goal was to enhance the osteogenic response to periosteal elevation by altering distraction parameters. We hypothesized that the apposition of the newly formed bone will be accompanied by anbolic remodeling of the pristine calvarial bone. Hence, the primary outcome was the amount of newly formed bone assessed histomorphometrically.

## Results

### Clinical observations

All animals recovered well and quickly after surgery. Minor swelling was occasionally observed after surgery, which resolved within a few days. Clinical examinations revealed uneventful healing in all other animals with normal behavior without impairment in their appearance and water and food intake. The distraction device was exposed in one animal (PE_1). No devices were lost during the observation period.

### Qualitative histological analysis

Signs of locally confined inflammatory reaction were observed in five animals: three animals (two LD and one sham group) on day 4 and two animals (LD group) on day 7 of the observation period. Histological analysis revealed signs of minor infection next to the distraction screw in nine animals: five animals (two PDO, one D_PP, one PP, and one PE_1) on day 17, one animal (PDO) on day 31, and three animals (DDP, PP, and PE_1) on day 45 of the observation period. Minute exposure of the device was observed in one animal (PP_D) on day 31 and one animal (PDO) on day 45 of the observation period. All the samples were included in the analysis.

Bone formation to different extents throughout the observation period was observed in all animals. The gap between the new bone and the distraction plate was occupied by loose connective tissue. The type of new bone was woven bone at the leading edge of the bone apposition. Signs of bone maturation were observed over time in all groups. The maximal values of newly formed bone for all groups with distraction plates were found in region of interest_2 (ROI_2, the higher part of the distraction gap), with comparable values observed in ROI_1 and ROI_3. The original calvarial bone mainly comprised compact bone with intervening marrow cavities. Bone marrow was present in the middle part of the calvaria and was generally more extended in ROI_2. The old calvarial bone and newly formed bone were occasionally connected to the bone marrow cavities and blood vessels, especially in ROI_2. When the calvarial bone was thin, the bone marrow was presented as small isolated islands or was completely absent, as sporadically observed in ROI_1. Differences were observed in bone modeling and remodeling between the groups.

### Latency period

The CNNB1 in the LD group was significantly overexpressed in the bone (+ 53% vs. sham) and periosteum (+ 65% vs. sham) on day 1 of the observation period (Table 1). Sost expression in the bone was also upregulated in the LD group on day 1 of the observation period (+ 429% vs. sham), reaching the significant effect for the device placement, healing period, and their interactions. The bone expression of CNNB1, Runx2, Sparc, ACP5, and BMP2 on day 4 of the observation period in the LD group was downregulated (− 62%, − 36%, − 36%, − 59%, and − 81%, respectively vs. sham). Periosteal expression of the ACP5 in the LD group was significantly upregulated, while Sost in the periosteum was not expressed (Table 1).

**Table 1:** Periosteal and bone gene expression during latency period by device placement and healing period. Significant values are in bold.

		Sham group (without device)			LD group (with device)			Two-way ANOVA			Pairwises comparisons		
	Gene Expression /Gapdh	1 day	4 days	7 days	1 day	4 days	7 days	device Effect	Healing Effect	Device x Healing	1 day	4 days	7 days
Periosteum	CNNB1	1.58 ± 0.03	2.5 ± 0.24	2.37 ± 0.75	2.43 ± 0.06	3.9 ± 0.53	1.03 ± 0.24	0.369	**0.007**	**0.012**	**0.000**	0.073	0.165
	Runx2	0.55 ± 0.08	2.37 ± 0.39	1.66 ± 0.62	0.91 ± 0.06	1.41 ± 0.37	1.39 ± 0.16	0.324	**0.016**	0.198	**0.025**	0.150	0.692
	Sox9	0.28 ± 0.1	0.83 ± 0.29	1.28 ± 0.86	0.26 ± 0.01	2.3 ± 0.37	1.61 ± 0.18	0.099	**0.014**	0.201	0.847	**0.034**	0.723
	Sparc	0.55 ± 0.05	2.44 ± 0.26	2.08 ± 0.97	0.14 ± 0.02	1.6 ± 0.24	1.31 ± 0.44	0.096	**0.008**	0.882	**0.001**	0.074	0.510
	ACP5	0.08 ± 0.01	0.25 ± 0.02	0.25 ± 0.09	0.34 ± 0.04	0.65 ± 0.33	1.67 ± 0.16	**0.000**	**0.001**	**0.005**	**0.004**	0.285	**0.001**
	BMP2	1.21 ± 0.07	2.04 ± 0.31	2.11 ± 0.84	0.24 ± 0.03	1.59 ± 0.45	1.33 ± 0.34	0.061	0.052	0.832	**0.000**	0.460	0.440
	Postn	0.46 ± 0.03	2.34 ± 0.34	1.99 ± 1.02	0.19 ± 0.04	0.97 ± 0.17	1.91 ± 0.35	0.158	**0.011**	0.361	**0.005**	**0.022**	0.944
	Sost	0 ± 0	0 ± 0	0 ± 0	0 ± 0	0 ± 0	0 ± 0	1.000	1.000	1.000			
	Coll1a	0.44 ± 0.06	2.43 ± 0.22	2.1 ± 0.91	0.26 ± 0.05	1.02 ± 0.15	1.86 ± 0.11	0.079	**0.003**	0.245	0.077	**0.006**	0.804
Bone	CNNB1	1.26 ± 0.33	1.84 ± 0.06	2.17 ± 0.63	2.35 ± 0.2	0.68 ± 0.04	1.62 ± 0.71	0.561	0.303	0.052	**0.049**	**0.000**	0.595
	Runx2	2.19 ± 0.35	1.59 ± 0.09	2.88 ± 0.5	2.38 ± 0.62	1.01 ± 0.09	1.3 ± 0.2	0.050	**0.046**	0.094	0.803	**0.012**	**0.042**
	Sox9	0.89 ± 0.32	1.31 ± 0.05	1.61 ± 0.52	1.57 ± 0.16	0.35 ± 0.03	0.9 ± 0.43	0.226	0.352	**0.048**	0.128	**0.000**	0.356
	Sparc	2.1 ± 0.13	1.5 ± 0.02	2.9 ± 0.6	1.76 ± 0.11	0.96 ± 0.05	1.09 ± 0.09	**0.001**	**0.023**	**0.031**	0.122	**0.001**	**0.041**
	ACP5	2.06 ± 0.22	1.42 ± 0.02	1.72 ± 0.24	0.62 ± 0.09	0.58 ± 0.11	1.64 ± 0.14	**0.000**	**0.004**	**0.004**	**0.004**	**0.002**	0.774
	BMP2	1.33 ± 0.36	1.38 ± 0.2	2.26 ± 0.74	0.6 ± 0.07	0.25 ± 0.02	0.85 ± 0.56	**0.008**	0.216	0.724	0.119	**0.005**	0.207
	Postn	2.03 ± 0.49	0.98 ± 0.15	2.24 ± 0.57	1.86 ± 0.3	1.12 ± 0.13	1.67 ± 0.17	0.490	**0.035**	0.596	0.779	0.507	0.385
	Sost	1.12 ± 0.36	0.47 ± 0.06	1.6 ± 0.49	5.93 ± 0.67	0.58 ± 0.19	1.73 ± 0.29	**0.000**	**0.000**	**0.000**	**0.003**	0.615	0.833
	Coll1a	0.44 ± 0.06	2.43 ± 0.22	2.98 ± 0.82	0.35 ± 0.06	1.19 ± 0.39	2.07 ± 0.27	**0.040**	**0.001**	0.364	0.318	0.051	0.349

**Figure 1:**
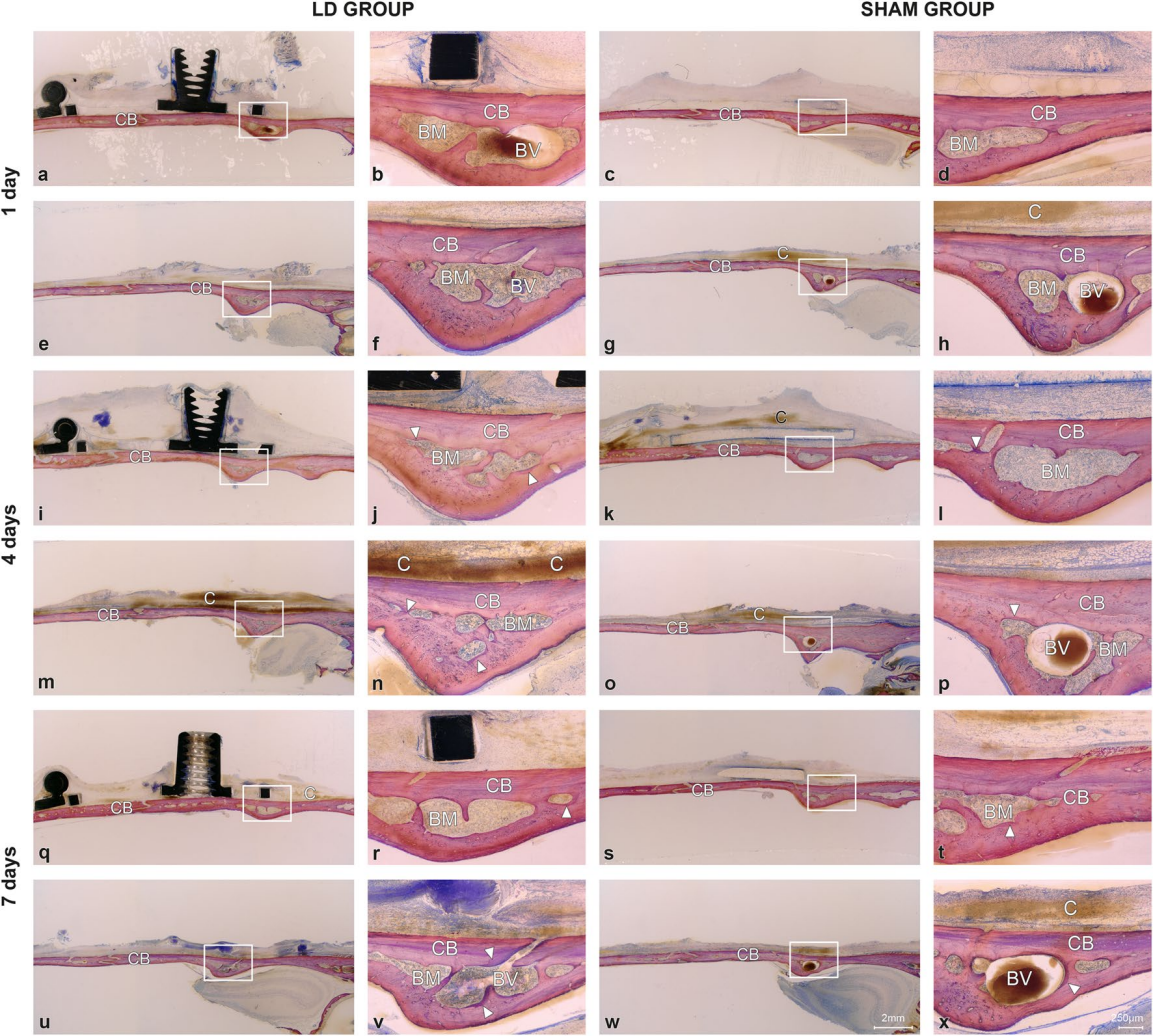
Representative transversal histological images of the calvarium illustrating sections in the midaxis (above) and outside the distraction plate (below) in the DC and Sham groups at 1-day, 4-day and 7-day observation periods (latency period). The boxed areas in overviews (left) are magnified (right), showing calvarial bone (CB) with bone marrow (BM) and blood vessels (BV). Signs of bone remodeling (arrowheads) are observed lining the bone marrow cavities at 4-day and 7-day observation periods. Remnants of blood clot (C) are visible at all three observation periods. Toluidine blue and fuchsin staining.

The swelling was more intense in the LD group than in the sham group on days 1 and 4 of the observation period and declined on day 7 (Fig. 1). A distinct periosteal layer was discernible in both groups and was sporadically disrupted in the LD group. Intensive coagulum formation was generally observed above the periosteum during the latency period, independent of device placement (Supplemental Fig. S1). Signs of minimal bone formation between the periosteum and the original bone surface were observed on day 4 of the observation period in both groups (Supplemental Fig. S2). The MBBF was consistently higher in the sham group than in the LD group, reaching significance for the newly formed woven bone (WB), bone marrow (BM), and total new bone area (TNB) on day 4 of the observation period (Table 2). Device placement, healing period, and their interaction negatively affected the MBBF. On day 7 of the observation period, patch-wise deposition of primary woven bone covered with osteoid and osteoblasts was observed in both groups (Supplemental Fig. S2). Osteoclasts were occasionally observed in the LD group at the sites where the device contacted the original bone surface. Minimal signs of bone turnover on days 4 and 7 of the observation periods were always associated with the presence of BM (Fig. 1, Supplemental Fig. S1). Device placement negatively affected relative calvarial bone marrow (R_CBM) on day 4 of the observation period.

**Table 2:** Morphometric parameters during latency period by device placement and healing period.

	Sham group (without device)			LD group (with device)			Two-way ANOVA			Pairwises comparisons		
Parameters	1 day	4 days	7 days	1 day	4 days	7 days	Device Effect	Healing Effect	Device x Healing Effect	1 day	4 days	7 days
WB	0.60 ± 0.36	2.77 ± 1.06	1.61 ± 0.98	0.49 ± 0.29	1.05 ± 0.31	1.27 ± 0.19	** < .001**	** < .001**	** < .001**	.353	** < .001**	.206
BM	0.00 ± 0.01	0.09 ± 0.08	0.69 ± 0.42	0.00 ± 0.00	0.03 ± 0.03	0.15 ± 0.16	** < .001**	** < .001**	** < .001**	**.042**	**.018**	** < .001**
TNB	0.61 ± 0.37	2.87 ± 1.05	2.31 ± 1.31	0.49 ± 0.29	1.08 ± 0.31	1.42 ± 0.33	** < .001**	** < .001**	** < .001**	.331	** < .001**	**.023**
R_COB	83.73 ± 5.08	80.71 ± 13.44	81.16 ± 4.16	83.73 ± 5.45	87.27 ± 3.62	84.42 ± 7.13	.405	.950	.510	.999	.437	.823
R_CBM	13.64 ± 4.41	13.85 ± 5.34	14.92 ± 3.63	13.56 ± 4.83	10.03 ± 3.42	11.15 ± 6.04	.**012**	.385	.216	.966	**.029**	.050
R_CNB	2.62 ± 1.30	5.42 ± 13.25	3.90 ± 1.31	2.69 ± 4.10	2.68 ± 0.95	4.41 ± 4.98	.900	.348	.501	.945	.079	1

Two-way ANOVA p-values for the effect of device, healing and their interaction for day 1, 4 and 7 post-surgery. Means ± SD are shown. WB = new woven bone; BM = new bone marrow; TNB = total new bone; R_COB = relative % of old calvarial bone to total calvarial bone; R_CBM = relative % of calvaril bone marrow to total calvarial bone; R_CNB = relative % of calvarial new bone to total calvarial bone.

Significant values are in [bold].

The micro-computed tomography (μCT) parameters varied between the groups. Cortical porosity (Ct.Po) and marrow volume (Ma.V) were lower in the LD group than in the sham group; however, none of the parameters showed a statistically significant difference.

### Day 17 of the observation period (end-activation)

The PP group demonstrated the highest CNNB1 bone expression (+ 106% vs. sham; Table 3) and CNNB1, Sparc, and Coll1a periosteum expression (+ 143%, + 32%, and + 82%, respectively, *vs.* sham; Table 4).

**Table 3:** Gene expression in bone during consolidation period.

Observation period	Gene Expression /Gapdh	PDO	DDP	PP_D	D_PP	PP	PE_1	Sham	*P*
17-day	CNNB1	2.11 ± 0.07	2.36 ± 0.22	1.4 ± 0.87	2.29 ± 0.29	2.34 ± 0.27	1.1 ± 0.46	1.47 ± 0.27	0.227
	Runx2	2.03 ± 0.22	2.01 ± 0.24	1.32 ± 0.64	2.34 ± 0.59	2.48 ± 0.73	1.79 ± 0.81	2.11 ± 0.14	0.807
	Sox9	0.93 ± 0.13	1.03 ± 0.29	1.04 ± 0.72	1.22 ± 0.23	1.43 ± 0.3	0.6 ± 0.22	0.9 ± 0.23	0.766
	Sparc	1.89 ± 0.26	1.75 ± 0.09	1.75 ± 0.97	2.37 ± 0.54	2.54 ± 1.06	1.7 ± 0.82	2.22 ± 0.15	0.947
	ACP5	1.58 ± 0.19	1.86 ± 0.42	0.89 ± 0.27	2.09 ± 0.49	1.43 ± 0.48	0.93 ± 0.4	0.67 ± 0.13	0.114
	BMP2	0.89 ± 0.09	1.6 ± 0.6	1.03 ± 0.69	1.35 ± 0.14	1.58 ± 0.21	1.44 ± 0.6	1.61 ± 0.36	0.853
	Postn	1.61 ± 0.16	1.54 ± 0.25	0.51 ± 0.18	1.92 ± 0.26	1.68 ± 0.49	1.67 ± 0.67	1.33 ± 0.46	0.305
	Sost	1.64 ± 0.14	1.37 ± 0.31	1.43 ± 0.95	2.51 ± 0.85	1.87 ± 0.78	1.83 ± 0.73	1.25 ± 0.36	0.853
	Coll1a	2.58 ± 0.58	2.32 ± 0.62	0.59 ± 0.16	3.63 ± 0.11	3.74 ± 0.49	1.65 ± 0.69	1.81 ± 0.27	**0.004**
31-day	CNNB1	0.74 ± 0.25	2.69 ± 1.19	1.81 ± 0.71	1.37 ± 0.49	4.29 ± 0.09	1.96 ± 0.51	2.05 ± 0.91	**0.034**
	Runx2	1.66 ± 0.62	3.9 ± 1.73	2.78 ± 0.1	1.37 ± 0.7	9.22 ± 1.67	1.61 ± 0.48	5.3 ± 2.14	**0.011**
	Sox9	0.52 ± 0.13	1.7 ± 0.83	2 ± 0.6	0.96 ± 0.4	3.58 ± 0.53	0.98 ± 0.21	1.52 ± 0.57	**0.030**
	Sparc	1 ± 0.39	3.06 ± 1.56	2 ± 0.15	1.15 ± 0.69	7.64 ± 0.82	1.25 ± 0.37	3.19 ± 1.1	**0.002**
	ACP5	1.37 ± 0.51	4.01 ± 1.75	3.3 ± 0.5	1.53 ± 0.94	6.55 ± 2.41	0.9 ± 0.25	1.01 ± 0.02	0.078
	BMP2	0.47 ± 0.24	3.3 ± 1.59	1.81 ± 0.96	1.26 ± 0.48	3.71 ± 0.83	1.21 ± 0.25	2.29 ± 0.72	0.113
	Postn	1.1 ± 0.44	1.54 ± 0.53	1.43 ± 0.11	0.49 ± 0.16	4.73 ± 1.58	1.33 ± 0.41	1.53 ± 0.36	**0.018**
	Sost	1.26 ± 0.57	2.31 ± 0.91	3.14 ± 1.17	0.54 ± 0.2	9.11 ± 2.55	1.74 ± 0.74	3.63 ± 2.58	0.06
	Coll1a	0.3 ± 0.02	0.94 ± 0.34	0.63 ± 0.1	0.4 ± 0.25	0.96 ± 0.49	1.43 ± 0.46	2.21 ± 0.79	**0.008**
45-day	CNNB1	3.52 ± 1.35	0.59 ± 0.19	2.03 ± 0.33	1.55 ± 0.07	2.01 ± 0.34	3.73 ± 1.14	4.88 ± 0.01	**0.009**
	Runx2	2.5 ± 0.84	0.55 ± 0.16	2.5 ± 0.92	1.21 ± 0.37	1.49 ± 0.44	2.67 ± 0.14	13.57 ± 0.27	**0.000**
	Sox9	1.74 ± 0.39	0.25 ± 0.16	1.75 ± 0.14	0.88 ± 0.4	1.19 ± 0.45	2.66 ± 1.32	3.06 ± 0.48	0.061
	Sparc	1.54 ± 0.3	0.36 ± 0.11	1.76 ± 0.67	0.89 ± 0.24	1.07 ± 0.37	1.45 ± 0.07	5.21 ± 0.18	**0.000**
	ACP5	1.01 ± 0.2	0.38 ± 0.21	2.39 ± 0.88	0.76 ± 0.09	0.7 ± 0.26	1.09 ± 0.14	1.47 ± 0.12	**0.036**
	BMP2	0.65 ± 0.07	0.62 ± 0.34	1.65 ± 0.35	0.72 ± 0.1	1.52 ± 0.31	2.71 ± 1.33	3.52 ± 0.15	**0.013**
	Postn	2.01 ± 0.23	0.46 ± 0.19	2.07 ± 0.99	0.39 ± 0.09	0.74 ± 0.36	2.1 ± 0.2	2.22 ± 0.21	**0.017**
	Sost	3.77 ± 2.37	0.31 ± 0.1	2.07 ± 0.77	0.96 ± 0.21	1.06 ± 0.24	3.06 ± 0.42	10.11 ± 0.47	**0.000**
	Coll1a	4.08 ± 0.7	0.59 ± 0.39	0.63 ± 0.11	1.02 ± 0.15	0.52 ± 0.19	0.36 ± 0.21	3.04 ± 0.01	**0.000**

Means ± SD are shown. PDO = 1 activation every 24 h for 7 days; PDP = 2 activations/1 relaxation every 12 h for 10 days; D_PP = 1 activation every 24 h for 7 days followed by 1 activation/1 relaxation every 12 h for 3 days; PDP = 1 activation/1 relaxation every 12 h for 3 days followed by 1 activation every 24 h for 7 days; PP = 1 activation/1 relaxation every 12 h for 10 days; PE_1 = 1 activation; Sham = flap elevation without device placement. 17 days = end-activation period; 31 days = mid-consolidation period; 45 days = late-consolidation period.

Significant values are in [bold].

**Table 4:** Gene expression in periosteum during consolidation period.

Observation period	Gene expression /Gapdh	PDO	DDP	PP_D	D_PP	PP	PE_1	Sham	*P*
17-day	CNNB1	3.76 ± 0.15	3.81 ± 1.06	0.92 ± 0.07	3.75 ± 0.41	4.3 ± 0.43	1.72 ± 0.38	1.77 ± 0.46	**0.002**
	Runx2	2.95 ± 0.56	0.84 ± 0.08	0.6 ± 0.2	2.54 ± 0.43	3.01 ± 0.32	1.91 ± 0.44	0.58 ± 0.23	**0.000**
	Sox9	1.19 ± 0.75	0.17 ± 0.05	0.12 ± 0.04	2.97 ± 1.79	2.14 ± 1.03	0.41 ± 0.16	0.09 ± 0.02	0.156
	Sparc	2.27 ± 0.12	2.4 ± 0.51	0.82 ± 0.2	2.74 ± 0.27	3.19 ± 0.43	1.25 ± 0.47	2.41 ± 0.85	**0.034**
	ACP5	1.53 ± 0.12	0.15 ± 0.04	0.24 ± 0.13	0.46 ± 0.11	0.6 ± 0.08	1.47 ± 0.44	0.17 ± 0.10	**0.000**
	BMP2	1.48 ± 0.24	1.67 ± 0.83	0.71 ± 0.09	2.25 ± 0.28	1.79 ± 0.38	1.71 ± 0.28	1.59 ± 0.39	0.343
	Postn	2.32 ± 0.61	1.27 ± 0.15	0.72 ± 0.21	2.02 ± 0.33	2.96 ± 0.17	1.78 ± 0.81	2.25 ± 0.88	0.155
	Sost	0 ± 0	0 ± 0	0 ± 0	0 ± 0	0 ± 0	0 ± 0	0 ± 0	0.492
	Coll1a	2.58 ± 0.58	2.32 ± 0.62	0.59 ± 0.16	3.37 ± 0.22	3.74 ± 0.49	1.65 ± 0.69	2.05 ± 0.67	**0.015**
31-day	CNNB1	0.74 ± 0.19	1.81 ± 0.75	1.38 ± 0.25	0.93 ± 0.52	1.96 ± 0.95	2.15 ± 0.58	3.03 ± 1.2	0.172
	Runx2	0.39 ± 0.15	1.31 ± 0.5	0.61 ± 0.22	0.33 ± 0.18	1.07 ± 0.38	1.93 ± 0.86	1.53 ± 0.66	**0.041**
	Sox9	0.05 ± 0.01	2.21 ± 2.03	0.12 ± 0.05	0.08 ± 0.04	0.14 ± 0.04	0.75 ± 0.25	0.33 ± 0.14	0.431
	Sparc	0.56 ± 0.07	1.25 ± 0.44	1.28 ± 0.24	0.73 ± 0.47	1.68 ± 0.85	1.54 ± 0.39	2.44 ± 1.06	0.199
	ACP5	0.17 ± 0.1	0.58 ± 0.19	0.15 ± 0.06	0.14 ± 0.07	0.2 ± 0.1	1.14 ± 0.55	0.15 ± 0.08	0.101
	BMP2	0.47 ± 0.11	2.75 ± 1.78	1.42 ± 0.38	0.72 ± 0.53	2.11 ± 0.95	1.71 ± 0.53	2.84 ± 1.19	0.459
	Postn	0.36 ± 0.03	1.21 ± 0.52	0.81 ± 0.21	0.47 ± 0.28	0.93 ± 0.29	1.83 ± 0.65	1.21 ± 0.48	**0.001**
	Sost	0 ± 0	0.11 ± 0.11	0 ± 0	0 ± 0	0 ± 0	0 ± 0	007 ± 0.07	0.152
	Coll1a	0.3 ± 0.02	0.94 ± 0.34	0.63 ± 0.1	0.4 ± 0.25	0.96 ± 0.49	1.63 ± 0.56	1.33 ± 0.63	**0.007**
45-day	CNNB1	6.09 ± 2.26	1.35 ± 0.68	1.77 ± 0.45	0.44 ± 0.12	1.81 ± 0.63	3.81 ± 1.04	4.86 ± 1.42	**0.033**
	Runx2	1.97 ± 0.56	0.65 ± 0.46	1.15 ± 0.14	3.9 ± 0.53	1.28 ± 0.55	0.45 ± 0.08	1.85 ± 0.57	**0.002**
	Sox9	2.79 ± 1.76	0.21 ± 0.08	0.32 ± 0.15	1.41 ± 0.37	0.52 ± 0.22	0.36 ± 0.06	0.28 ± 0.08	0.147
	Sparc	2.38 ± 0.83	0.71 ± 0.39	0.98 ± 0.11	2.3 ± 0.37	0.65 ± 0.21	0.42 ± 0.33	2.55 ± 1	0.050
	ACP5	0.82 ± 0.16	0.27 ± 0.2	1.17 ± 0.42	1.6 ± 0.24	0.91 ± 0.4	0.24 ± 0.03	1.25 ± 0.88	0.272
	BMP2	3.58 ± 2.09	0.41 ± 0.12	1.67 ± 0.39	0.65 ± 0.33	0.71 ± 0.33	0.72 ± 0.14	5.49 ± 1.91	**0.038**
	Postn	2.8 ± 1.52	0.45 ± 0.29	0.76 ± 0.03	0.97 ± 0.17	0.52 ± 0.18	0.43 ± 0.19	1.87 ± 0.68	0.152
	Sost	0 ± 0	0 ± 0	0.85 ± 0.44	0 ± 0	0 ± 0	0 ± 0	2.11 ± 0.11	**0.000**
	Coll1a	1.66 ± 0.69	0.59 ± 0.39	0.55 ± 0.06	1.02 ± 0.15	0.52 ± 0.19	0.29 ± 0.15	1.24 ± 0.53	0.203

Means ± SD are shown. PDO = 1 activation every 24 h for 7 days; PDP = 2 activations/1 relaxation every 12 h for 10 days; D_PP = 1 activation every 24 h for 7 days followed by 1 activation/1 relaxation every 12 h for 3 days; PDP = 1 activation/1 relaxation every 12 h for 3 days followed by 1 activation every 24 h for 7 days; PP = 1 activation/1 relaxation every 12 h for 10 days; PE_1 = 1 activation; Sham = flap elevation without device placement. 17 days = end-activation period; 31 days = mid-consolidation period; 45 days = late-consolidation period.

Significant values are in [bold].

**Figure 2:**
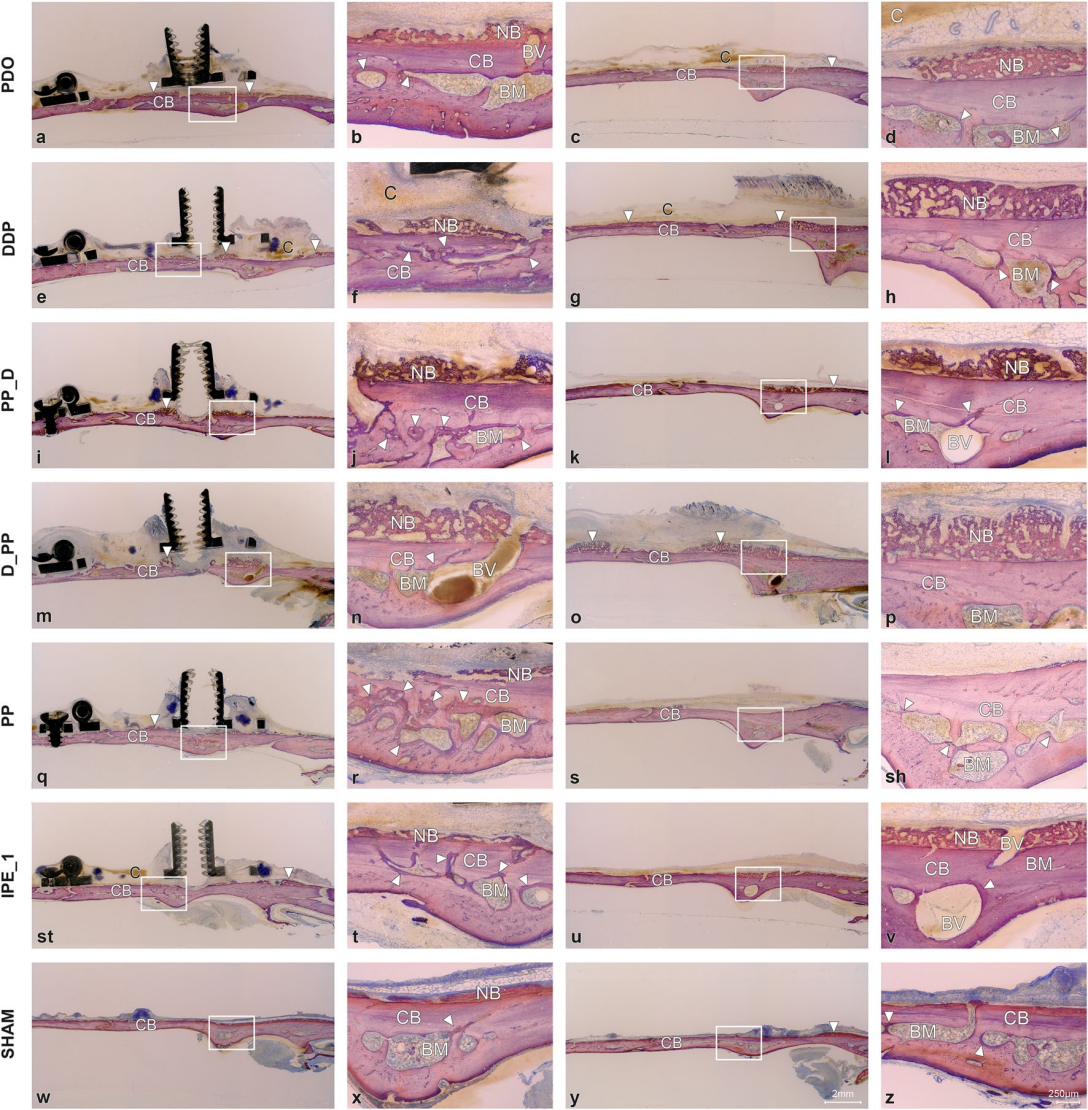
Representative transversal histological images of the calvarium illustrating sections in the midaxis (left) and outside the distraction plate (right) at 17-day observation period (end-distraction). The overviews demonstrating minor new bone apposition (arrowheads) on top of the original, calvarial bone (CB). Remnants of blood clot (C) are sporadically observed. The boxed areas in overviews are magnified, showing signs of bone remodeling (arrowheads) adjacent to the bone marrow (BM). Blood vessels (BV) are connecting calvarial bone with newly formed woven bone (NB). Toluidine blue and fuchsin staining.

**Figure 3:**
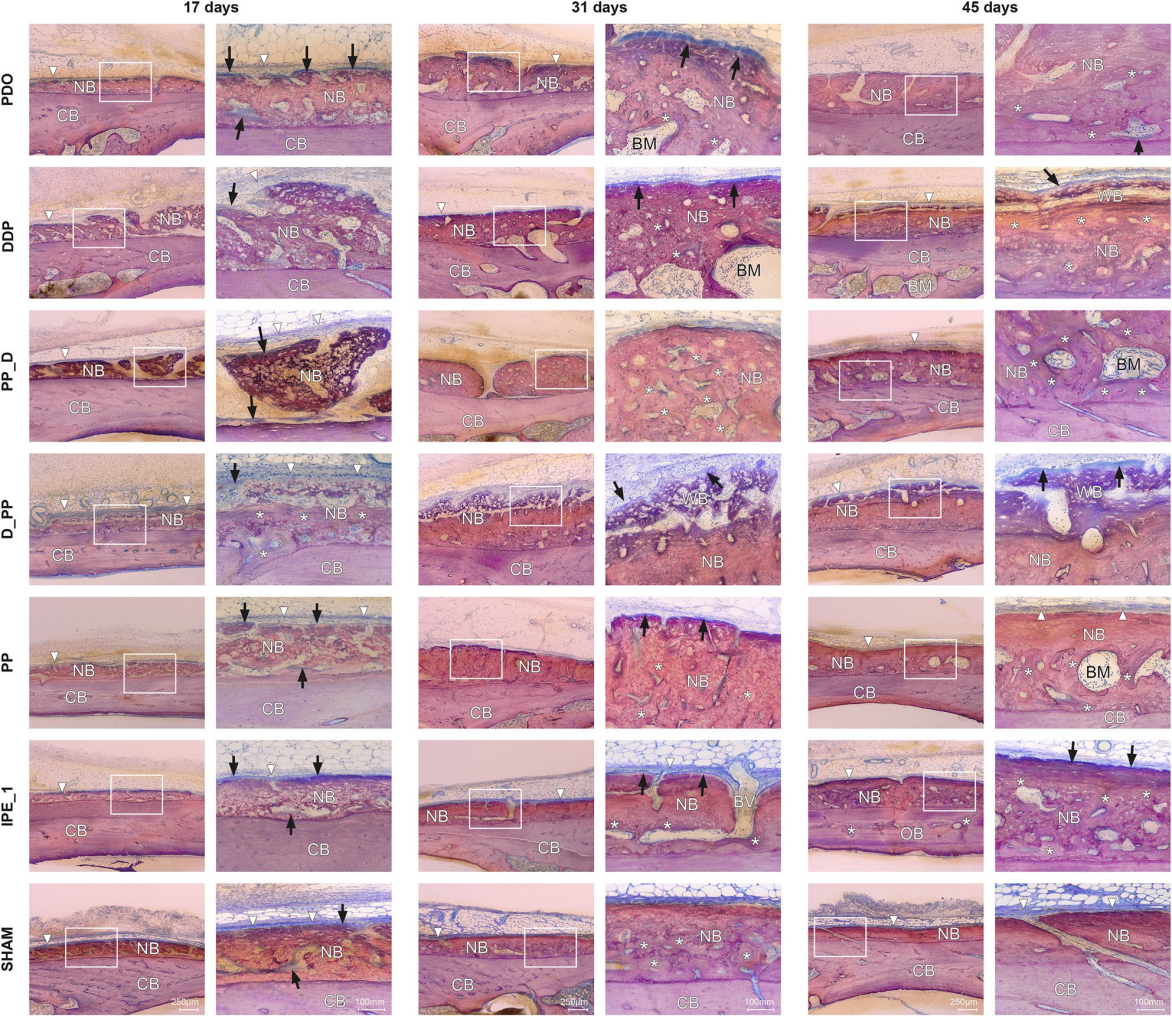
Representative transversal histological images of the calvarium illustrating new bone (NB) apposition on top of the original, calvarial bone (CB) in ROI_3 outside the distraction plate at 17-day, 31-day and 45-day observation periods. Newly formed bone is covered with the periosteum (arrowheads). The boxed areas (left) are magnified (right). New bone at 17-day observation period is mainly composted of the woven bone covered with the osteoid (arrows). At the 31-day observation period, newly formed bone is reinforced by parallel-fibred bone (*) and contain bone cavities with immature bone marrow (BM) and blood vessels (BV). New dense, lamellar bone (*) was observed in all groups at the 45-day observation period. A clear gradient in bone maturation was evident especially in the D_PP group, with woven bone (WB) indicative of ongoing bone formation.

**Figure 4:**
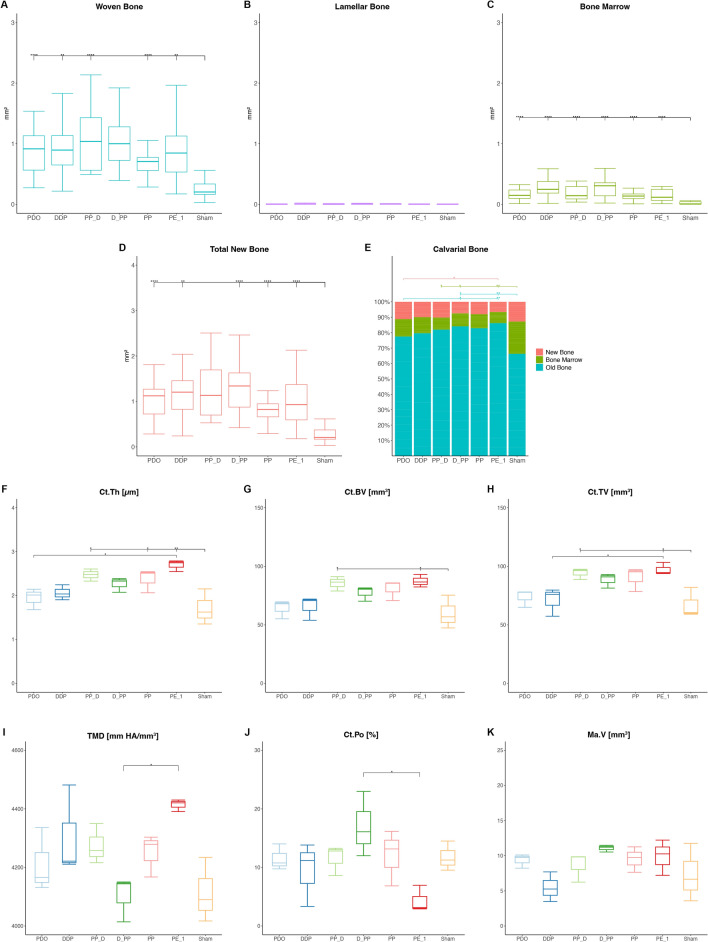
Effects of periosteal pumping on bone modeling and remodeling at 17-day observation period (end-activation). ROI_Sum was measured as a sum of ROI_1, ROI_2 and ROI_3. (**A**–**D**) Histomorphometric data of area parameters in the newly formed bone were analysed for woven bone (WB, mm^2^), lamellar bone (LB, mm^2^), bone marrow (BM, mm^2^) and total new bone (TNB, mm^2^). (**E**) The area fraction (%) of new bone (R_CNB = CNB/TCB), bone marrow (R_CBM = CBM/TCB) and old bone (R_COB = OB/TCB) were measured in the calvarial bone. (**F**–**K**) Micro-CT analysis of calvarias composed of newly formed bone and the original, calvarial bone. Quantification of cortical bone parameters showing cortical thickness (Ct.Th), cortical bone volume (Ct.BV), cortical tissue volume (Ct.TV), tissue mineral density (TMD), cortical porosity (Ct.Po) and marrow volume (Ma.V). Data are presented as box plots with median, means and interquartile ranges. Statistical analysis was performed by Kruskal–Wallis test with Dunn-Bonferroni’s adjustment (**A**–**E**) and one-way ANOVA followed by Tukey’s multiple comparison test (**F**–**K**). *p < 0.05, **p < 0.01 ***p < 0.001, ****p = 0.000.

The newly formed bone comprised woven bone with bone cavities (Fig. 2). A contiguous layer of new bone was sporadically reinforced by the parallel-fibered bone (Fig. 3). Signs of calvarial bone resorption and apposition were observed at the sites where the tip of the distraction screw touched the calvarial bone surface (Supplemental Fig. S3). Orbicular bone structures facing soft tissues with many osteoblasts and osteoids indicate ongoing bone formation. The greatest MBBF was observed in the PPD, PP_D, and D_PP groups (Fig. 2), with the D_PP group showing a highly significant difference for NB, BM, and TNB compared to the sham group (Fig. 4). The groups with plate elevation (W/PE) demonstrated higher WB (*p* ≤ 0.05), TNB (*p* = 0.047), and relative calvarial new bone (R_CNB, *p* = 0.04) values than the groups without plate elevation (Wo/PE), whereas no significant effect was observed for the pumping protocol. The PP group demonstrated the lowest MBBF values among all groups with the plate. The sham group demonstrated minimal MBBF and the highest R_CNB and R_CBM values (Fig. 4e). More intensive bone remodeling was observed in all groups compared to the latency period, especially in ROI_2.

The W/PE group demonstrated higher cortical thickness (Ct.Th, *p* = 0.017) and cortical tissue volume (Ct.TV, *p* = 0.049) than the Wo/PE group. The PP_D and PE_1 groups had the highest Ct.Th, cortical bone volume (Ct.BV), and Ct.TV values (Fig. 4). Furthermore, an inverse relationship was detected in the PE_1 and D_PP groups for tissue mineral density (TMD) and Ct.Po values, with the lowest and highest values, respectively. The pumping protocol did not significantly affect the µCT parameters.

### Day 31 of the observation period (mid-consolidation)

The highest expression of most bone genes was detected for the PP group, reaching significance for CNNB1, Runx2, Sox9, Sparc, and Postn (+ 109%, + 73%, + 135%, + 139%, and + 209%, respectively, vs. sham; Table 3). Periosteal gene expression was downregulated in the test groups, with the lowest Runx2 expression in the D_PP group (-78% vs. sham) and the lowest Postn and Coll1a expression in the PDO group (− 70% and − 77%, respectively, vs. sham; Table 4).

**Figure 5:**
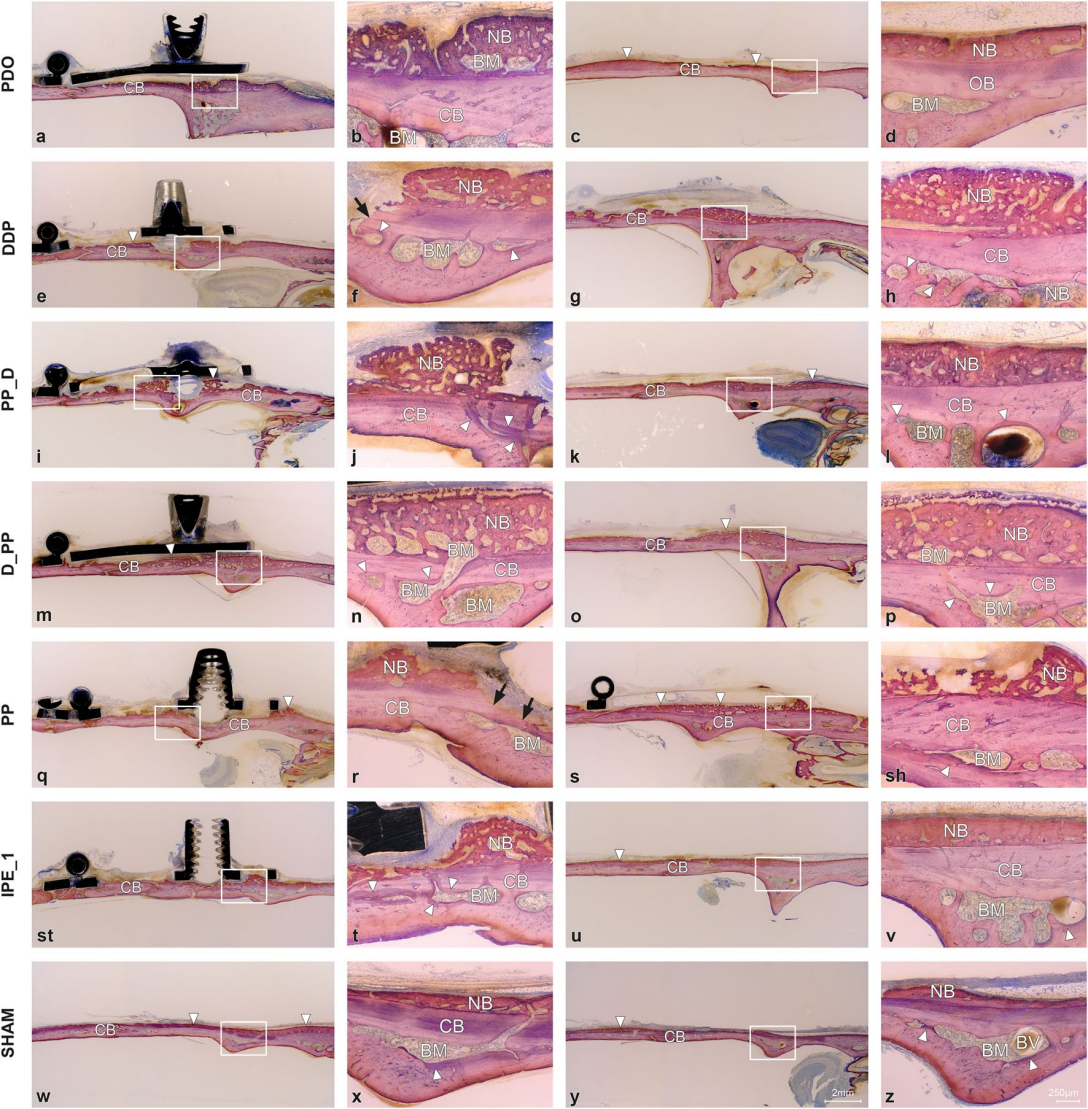
Representative transversal histological images of the calvarium illustrating sections in the midaxis (left) and outside the distraction plate (right) at 31-day observation period (mid-consolidation). The overviews showing bone formation (arrowheads) on the original, calvarial bone (CB). The boxed areas in overviews are magnified, demonstrating bone marrow (BM) and blood vessels (BV). Signs of bone remodeling (arrowheads) are seen next to the bone marrow. Bone resorbtion is observed at sites where distraction rod was in contact with the calvarial bone (arrows). Occasionally, bone marrow is intervening between the calvarial bone and newly formed bone (NB). Toluidine blue and fuchsin staining.

**Figure 6:**
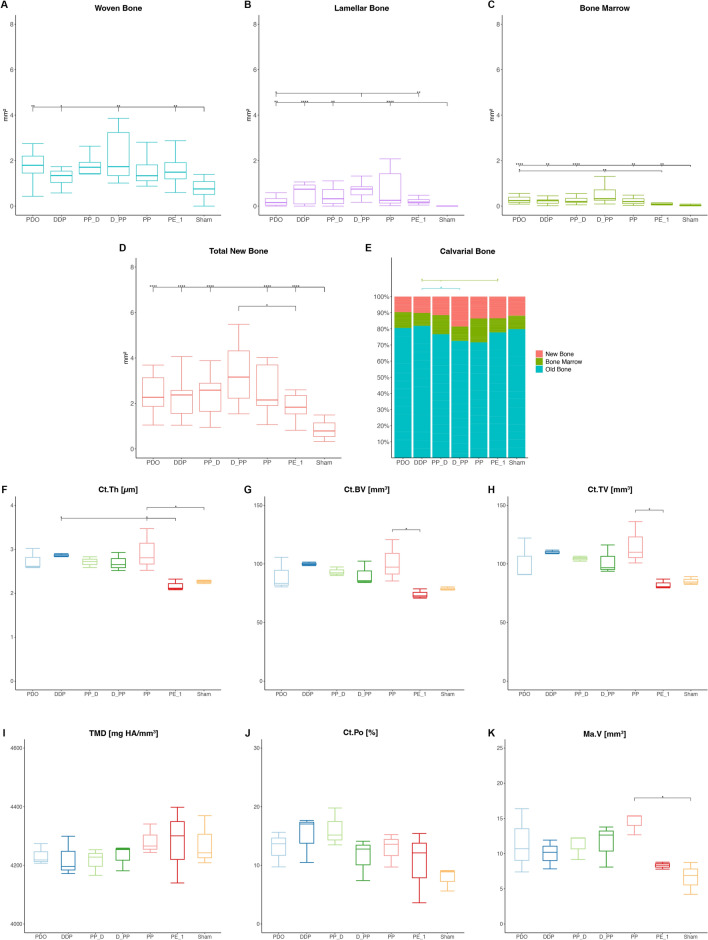
Effects of periosteal pumping on bone modeling and remodeling at 31-day observation period. ROI_Sum was measured as a sum of ROI_1, ROI_2 and ROI_3. (**A**–**D**) Histomorphometric data of area parameters in the newly formed bone were analysed for woven bone (WB, mm^2^), lamellar bone (LB, mm^2^), bone marrow (BM, mm^2^) and total new bone (TNB, mm^2^). (**E**) The area fraction (%) of new bone (R_CNB = CNB/TCB), bone marrow (R_CBM = CBM/TCB) and old bone (R_COB = OB/TCB) were measured in the calvarial bone. (**F**–**K**) Micro-CT analysis of calvarias composed of newly formed bone and the original, calvarial bone. Quantification of cortical bone parameters showing cortical thickness (Ct.Th), cortical bone volume (Ct.BV), cortical tissue volume (Ct.TV), tissue mineral density (TMD), cortical porosity (Ct.Po) and marrow volume (Ma.V). Data are presented as box plots with median, means and interquartile ranges. Statistical analysis was performed by Kruskal–Wallis test with Dunn-Bonferroni’s adjustment (**A**–**E**) and one-way ANOVA followed by Tukey’s multiple comparison test (**F**–**K**). *p < 0.05, **p < 0.01 ***p < 0.001, ****p = 0.000.

The newly formed woven bone in all the groups contained bone cavities with immature bone marrow reinforced with parallel-fiber bone (Fig. 5). A gradient in bone maturation was evident, with more mature bone moving towards the old calvarial bone. Osteoid-lined bone marrow cavities were present along the woven bone surface. Bone resorption sites were recognizable at contact sites between the distraction rod and bone surface, with intensive bone formation (Fig. 5, Supplemental Fig. S3). Fine orbicular structures towards the skin indicated intensive bone formation (Fig. 3). The W/PE group had more BM (*p* = 0.020) than the Wo/PE group, whereas the groups with pumping protocol (W/PP) had more BM (*p* = 0.024), new lamellar bone (LB, *p* = 0.008), and TNB (*p* = 0.042) than the groups without pumping protocol (Wo/PP). The D_PP group demonstrated the most intense signs of bone formation (Fig. 5), with the highest WB, LB, BM, TNB, and R_CNB values and the lowest relative calvarial old bone R_COB value (Fig. 6).

Plate elevation showed no significant effect on the µCT parameters. Except for TMD, all µCT parameters were significantly higher in the W/PP group than in the Wo/PP group, reaching significance for Ct.Th (*p* = 0.030), Ct.BV (*p* = 0.018), and Ct.TV (*p* = 0.020). Furthermore, the PP group had higher Cr.Th, Ct.BV, and Ct.TV values than the PE_1 group and Cr.Th and Ma.V values than the sham group (Fig. 6).

### Day 45 of the observation period (end-consolidation)

The Coll1a bone expression was highest in the PDO group (+ 34% vs*.* sham). Nevertheless, bone genes were generally downregulated compared to those in the sham group (Table 3). Furthermore, Sost was highly expressed in the sham group. CNNB1 and BMP2 periosteum expression was downregulated in the test groups, being lowest in the D_PP and DDP groups (− 91% and − 92%, respectively, vs. sham; Table 4). Sost expression was significantly upregulated in the sham group.

**Figure 7:**
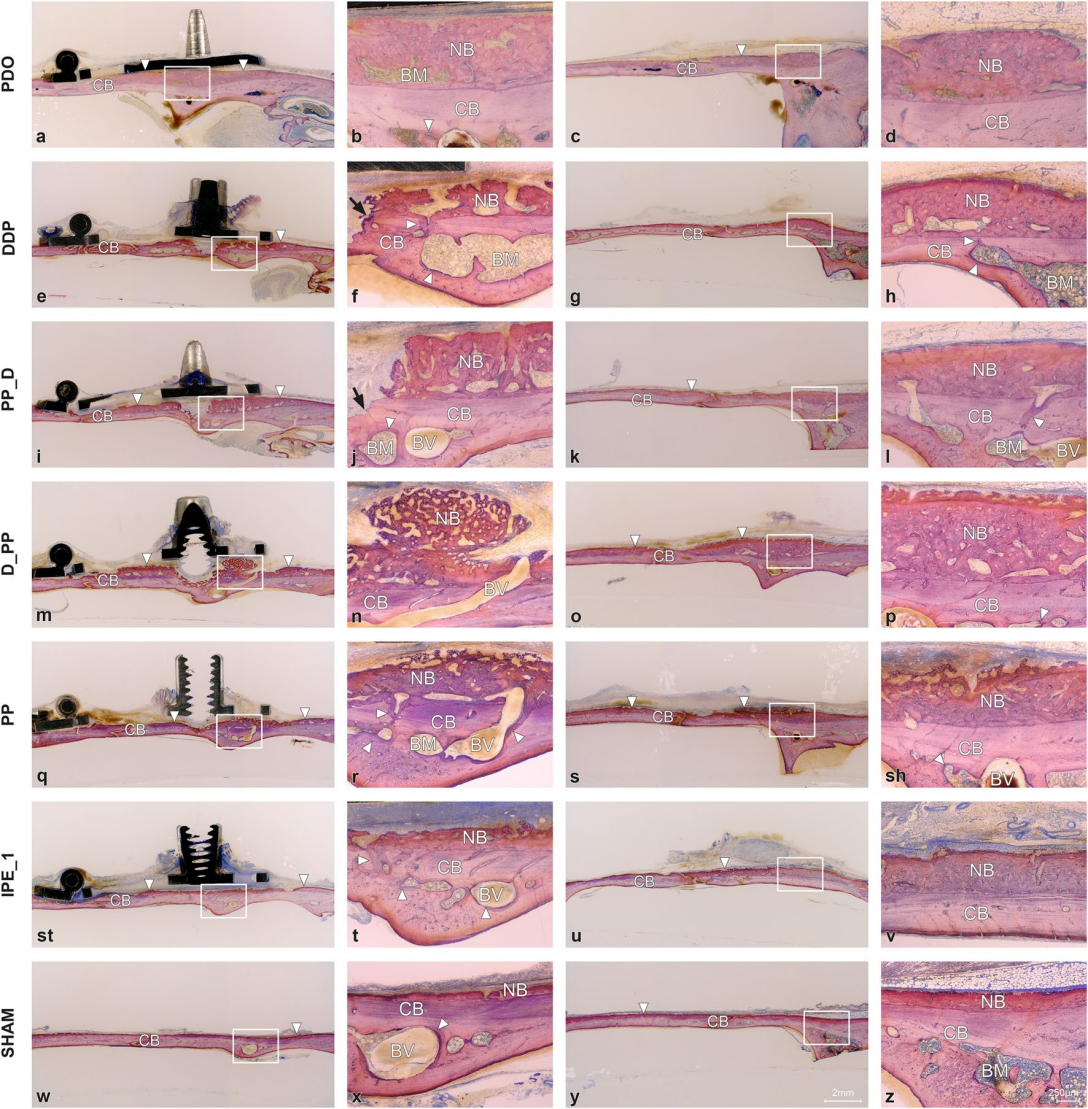
Representative transversal histological images of the calvarium illustrating sections in the midaxis (left) and outside the distraction plate (right) at 45-day observation period (end-consolidation). The overviews demonstrating newly formed bone (arrowheads) on top of the original, calvarial bone (CB). The boxed areas in overviews are magnified, showing signs of bone remodeling (arrowheads) adjacent to the bone marrow (BM) and blood vessels (BV). Occasionally, blood vessels are connecting calvarial bone with newly formed bone (NB). Resorbtion of the calvarial bone (arrows) is seen at sites in contact with the distraction rod. Toluidine blue and fuchsin staining.

**Figure 8:**
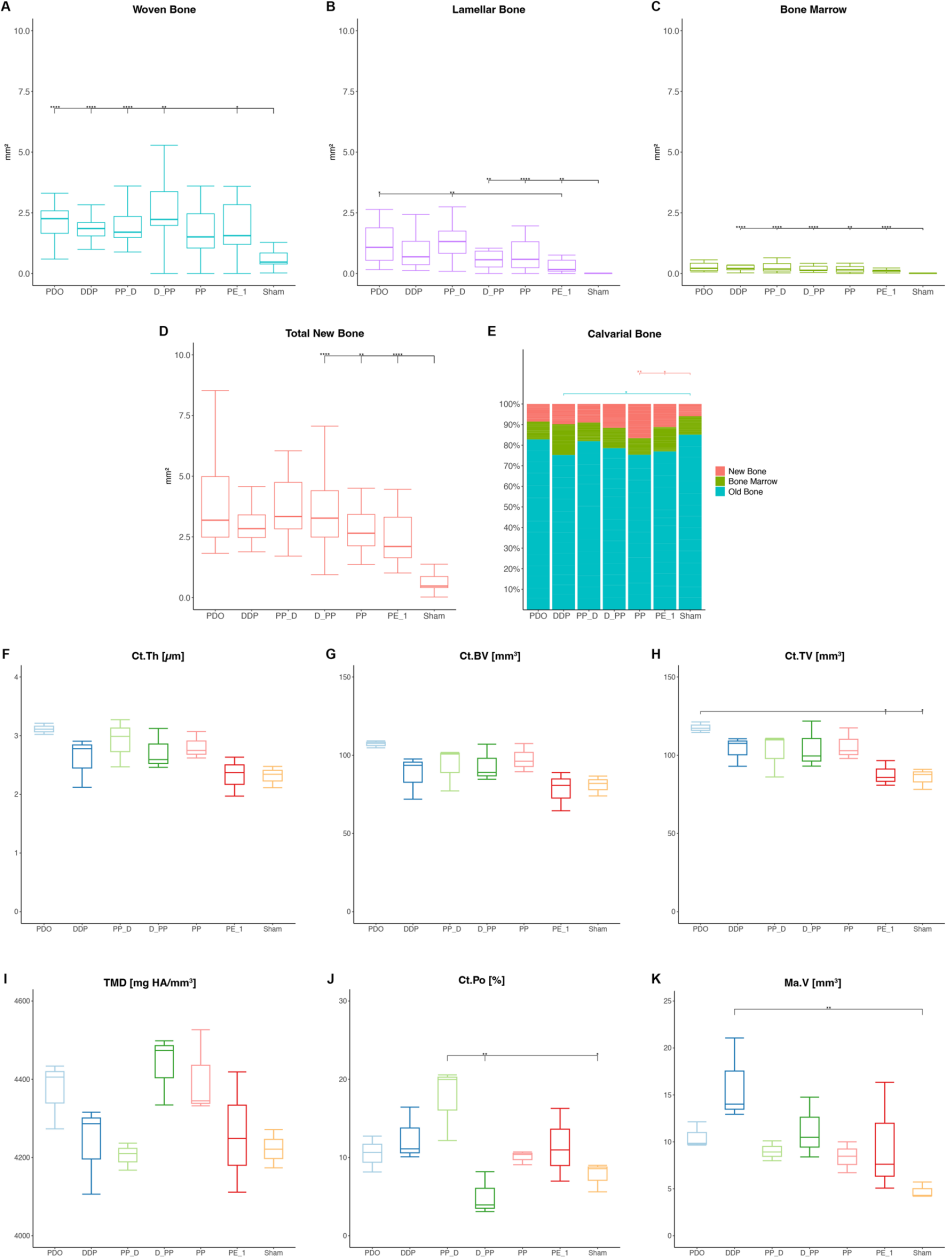
Effects of periosteal pumping on bone modeling and remodeling at 45-day observation period. ROI_Sum was measured as a sum of ROI_1, ROI_2 and ROI_3. (**A**–**D**) Histomorphometric data of area parameters in the newly formed bone were analysed for woven bone (WB, mm^2^), lamellar bone (LB, mm^2^), bone marrow (BM, mm^2^) and total new bone (TNB, mm^2^). (**E**) The area fraction (%) of new bone (R_CNB = CNB/TCB), bone marrow (R_CBM = CBM/TCB) and old bone (R_COB = OB/TCB) were measured in the calvarial bone. (**F**–**K**) Micro-CT analysis of calvarias composed of newly formed bone and the original, calvarial bone. Quantification of cortical bone parameters showing cortical thickness (Ct.Th), cortical bone volume (Ct.BV), cortical tissue volume (Ct.TV), tissue mineral density (TMD), cortical porosity (Ct.Po) and marrow volume (Ma.V). Data are presented as box plots with median, means and interquartile ranges. Statistical analysis was performed by Kruskal–Wallis test with Dunn-Bonferroni’s adjustment (**A**–**E**) and one-way ANOVA followed by Tukey’s multiple comparison test (**F**–**K**). *p < 0.05, **p < 0.01 ***p < 0.001, ****p = 0.000.

Woven bone formation with osteoids and numerous osteoblasts was observed beneath the distraction plate (Figs. 3 and 7). Bone resorption with Howship's lacunae and osteoclasts was still observed in the distraction screw region, with bone formation occurring next to the resorption sites (Fig. 7, Supplemental Fig. S3). The new bone layers in the three control groups were more mature. The new bone outside the plate consisted mainly of lamellar bone, cavities with mature bone marrow, and even surface contours without major signs of resorption (Fig. 3). The extent of bone formation among the groups with plate elevation was similar, reaching statistical significance for TNB, WB, and LB compared to the sham group (Fig. 8). The W/PE group showed significantly higher LB (*P* = 0.004), BM (*p* = 0.032), TNB (*p* = 0.005), and R_CNB (*p* = 0.003) values than the Wo/PE group, whereas no significant effect was detected for the pumping protocol. Sham group showed higher R_COB and lower R_CNB values than those in the PP group (Fig. 8). The highest R_CNB values in the PP group were also detected in the ROI_MS (Supplemental Fig. S4).

No significant effect was detected for plate elevation or pumping protocol on the µCT parameters. The PDO group showed the highest Ct.Th, Ct.BV, and Ct.TV values, which significantly differed for the Ct.TV in the PE_1 and sham groups (Fig. 8).

## Discussion

The present study's results indicate that the pumping protocol before the gradual elevation of the periosteum enhanced the MBBF at the mid-consolidation period, and without periosteal elevation the RBBF at the end-consolidation period. This, in turn, means that the hypothesis of the present study was confirmed. The space provided by the distraction plate positively affected the MBBF, as all groups with elevated plate had similar amounts of newly formed bone at the end-consolidation period. The model used in the present study is complex, as the process of periosteal wound healing is conditioned by mechanical stimuli.

Placing the distraction device during surgery disturbed the local environment. Device placement, healing period, and interaction significantly affected all three histomorphometric parameters. The upregulation of CNNB1 in the LD group one day after surgery suggests the activation of the Wnt/β-catenin signaling pathway, essential in regional anabolic response to loading^29^. Device placement, healing period, and interaction significantly affected the expression of Sost in bone, a potent inhibitor of the Wnt/β-catenin pathway. Sost expression is specific to terminally differentiated cells embedded within a mineralized matrix^30,31^. Sost binds to the first β-propeller in the extracellular domain of LRP5 and LRP6 and interferes with the formation of the LRP/Wnt/FZD heterotrimer. The expression of CNNB1 in the bone was significantly downregulated in the LD group on day 4 of the observation period. Furthermore, device placement negatively affected the expression of other osteogenic genes and all MBBF parameters on day 4 of the observation period. Sost has been identified as a key regulator of bone formation that inhibits osteoblast differentiation^32^ and stimulates osteoclast differentiation in a RANKL-dependent manner^33^. Sost expression in the periosteum was not detected in any of the samples. This was expected, as Sost is an osteocyte-specific protein^34^.

At the end of the activation period, all groups with device placement showed an increased MBBF compared to the sham group. MBBF depends mainly on the ability of the lining cells to form new bone upon stimulation with mechanical forces. Manipulation of the distraction plate significantly upregulated the periosteal expression of CNNB1, RunX2, and Sparc in the periosteum and Coll1a expression in the bone and periosteum, reaching the highest levels in the PP group. Maintaining contact between the periosteum and the underlying bone seems to play an important role in the upregulation of genes in the periosteum. However, the expression of RunX2 was upregulated in the PE_1 group. Mechanical stimuli are key determinants of bone mass; even low-magnitude mechanical stimulation has an anabolic effect on bones^35^. The effected movement and loading of the distraction plate was not controlled, as the manipulation of the distraction rod was performed manually. The instability of the distraction plate may also explain the higher MBBF values in the PE_1 group than in the sham group. In contrast, the sham group showed more R_CNB than the PE_1 group. Furthermore, the sham group showed the highest bone marrow area within the calvaria. Histologically observed signs of bone turnover were always related to the presence of the bone marrow. Decreased bone marrow may indicate adaptation of skeletal tissues to changes in the mechanical environment^36^. However, MBBF activation may be accompanied by RBBF prevention. This seemingly paradoxical observation in groups with plate elevation may be explained by the balance of bone formation and resorption at the periosteal and endosteal surfaces^37,38^. Alterations in cortical envelopes upon loading can accelerate the modeling of periosteal bone formation and endocortical resorption drift^39,40^. However, the highest Ct.Po in the D_PP group may be related to increased periosteal apposition and intracortical and endocortical remodeling^41^.

The PP group showed the highest expression of all evaluated genes in the bone at day 31 of the observation period. Thus, the importance of intimate contact between the periosteum and the underlying bone is confirmed for gene expression in the bone. The protocol in the PP group may have initiated Frost's regional acceleratory phenomenon that enhances all ongoing regional processes, modeling and remodeling, and healing of hard and soft tissues^42^. Following the initial activation, there is a subsequent propagation of the "load signal" to adjacent osteocytes, connected to the decreased expression of the Wnt ligand inhibitors in the cells that subsequently activate β-catenin signaling^29^. Deeply embedded osteocytes in the mid-cortical region are the first to be activated by a biochemical load signal generated from the bone surface^29^. The expression of CNNB1, Postn, and Sost in the bone was upregulated in the PP group without reaching significance for Sost; possible reasons for this may be the limited number of samples per group and the healing period. Nonetheless, Postn has a dual capacity to inhibit Sost expression and directly induce Wnt-b-catenin activation^43^. Furthermore, Postn mediates local MBBF and RBBF processes in response to fatigue loading^44^. As bone resorption decreased and bone formation relatively increased at day 31, the positive balance of local bone turnover increased RBBF^39^. The D_PP group, but not the PP group, exhibited a dual effect on both RBBF and MBBF. A possible explanation for this discrepancy in the lowest gene expression may be the time necessary for the progression of bone turnover as observed histologically. MBBF can be estimated as a part of the extensive RBBF, where a new bone matrix is formed over the peripheral area of the previous resorption lacunae, such as spilling over^45^.

On day 45 of the observation period**,** all groups with plate elevation levelled the TNB values of the D_PP achieved on day 31. The same plate elevation distance in these groups confirms the importance of space maintenance over a longer healing period^46^. Bone expression of Sost was downregulated in all groups compared to that in the sham group. Osteocytes respond to mechanical loading by decreasing sclerostin production^36,47^, which is increased by unloading^47^. An elevated level of Sost contributes to the absence of callus formation 30 days after fatigue^44^. Furthermore, Sost was expressed in the periosteum of the sham group. Other than osteocytes, several cell types can express *Sost*^34^. The minimal RBBF and MBBF values confirm that the sham group reached a steady state of bone homeostasis^48^. The highest RBBF was observed in the PP group two weeks after gene expression was induced, as previously observed in the D_PP group. The delay in RBBF compared to MBBF may be explained by the cortical bone of the calvaria with a small amount of bone marrow (without bone trabeculae), which differs in response to mechanical loading compared to the trabecular bone^39^. The highly significant difference achieved in the PP group suggests that considering specific indications, PP may be successfully performed to enhance both RBBF and MBBF, with the plates maintaining contact with the original bone. The lowest Ct.Po in the D_PP group indicated that fused lacunae were eventually replaced by a larger volume of a newly formed matrix that underwent primary and slower secondary mineralization^49^. Periosteal woven bone formation aids in rapid recovery from 15 to 30 days after fatigue loading^44^. The bone cells in the pumping groups on day 45 of the observation period may still be in the transient stage, as indicated by the presence of woven bone on the leading front of bone apposition^46^. Longer observation periods may be required to complete the remodeling process through the feedback loop of the mechanostat.

A simple and effective treatment approach is required for patients with extended bone deficiency. Endogenous stimulation of the MBBF and RBBF by the pumping protocol suggests a possible therapeutic role for the mechanically stimulated periosteum, avoiding the need for exogenous growth factors or stem cells. However, the wound dehiscence in the periosteal distraction remain the risk for unfavorable wound healing. Signs of exposure of the distraction plate were observed in the present study; nevertheless, the distraction seemed to be advantageous for the absence of major infection. Maximizing the activity of intrinsic growth factors may have significant clinical relevance as an alternative to more complex bone regeneration procedures. Prior to clinical application, several limitations should be still addressed. Detection of relevant proteins within the distraction gap utilizing immunohistochemistry or in situ hybridization will be necessary to confirm the RT-PCR results and address the knowledge gaps across the repair window. Analysis of an alternative mechanism involving crosstalk with other signaling pathways is warranted for a more comprehensive understanding of the repair processes. Since age imposes a significant variation on bone properties, additional studies are required beyond the young adult stage.

## Conclusions

The findings from this complex model of bone healing confirm the fundamental rule that mechanical stimulation can govern endogenous bone formation. Elevation of the periosteum from the bone surface significantly affected the MBBF and RBBF on days 17 and 45 of the observation period and the inclusion of the pumping protocol on day 31. Furthermore, the impact of PP on the osteogenic process depended on the relationship between the periosteum and the underlying bone surface. Maintenance of contact between the periosteum and the bone upregulated gene expression and RBBF, whereas periosteal elevation from the bony surface significantly enhanced MBBF. The link between Wnt pathway activation and mechanically induced RBBF and MBBF was confirmed, at least in part, via the downregulation of Sost.

## Material and methods

### Animals

All methods were carried out in accordance with the guidelines of the Institutional Animal Care Committee and reported in compliance with the ARRIVE guidelines. The study protocol was approved by the Committee for Animal Research, State of Bern, Switzerland (approval no. 31/18). One hundred and sixty-two 14-week-old male Wistar rats were acclimated for 14 days, housed in a room with an adjusted climate (temperature, 22–24 °C ± 2 °C; humidity, 30–60% ± 5%) and special sun-substitution ultraviolet light (12 h photoperiod, 6 h–18 h) without excessive or surprising noise. Three rats were housed in individually ventillated cages and fed a standard rodent diet and water ad libitum.

### Study design

Two groups of animals (N = 36, n = 18/group) were observed during latency period, with device placement (LD group) and without device placement (sham group). The animals of both groups were euthanized after one (n = 6/group), four (n = 6/group), and seven days (n = 6/group) following surgery. Seven groups of animals (N = 126, n = 18/group) were subjected to the different treatment modalities, and euthanized 17 (end-activation, n = 6/group), 31 (mid-consolidation, n = 6/group), and 45 days (end-consolidation period, n = 6/group) after surgery. Animals were randomized using a software package with a systematic randomization protocol (www.randomization.com). Potential confounders were not specifically controlled.

Sample size was calculated with an a-priori power model a level of significance a = 0.05 and error ß = 0.20 (power 1 − ß = 0.80), a peri-operatory risk of about 5% and a generic inter-individual variability. According to the power calculation, total sample size of 3 animals per subgroup were required to be 80% sure that the limits of a two-side 95% confidence interval will exclude a difference between the treatment modalities of more than 5%.

### Anesthesia and surgery

**Figure 9:**
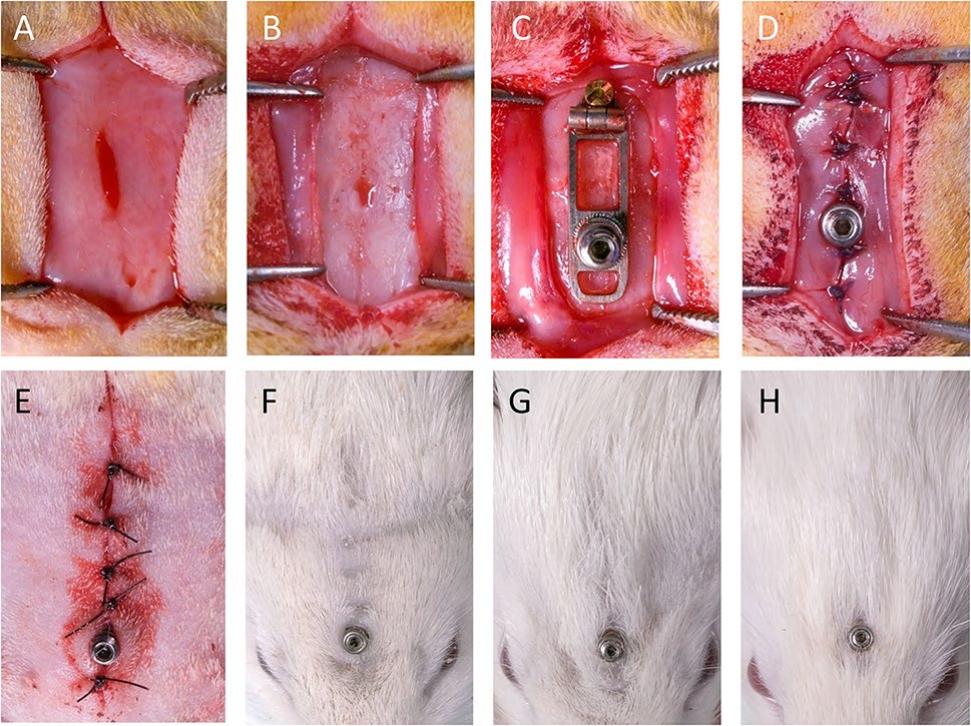
Intraoperative view of the surgical area following flap elevation (**A**). The periosteum was elevated (**B**) and the distraction device with perforated plate placed (**C**). The periosteum (**D**) and the skin (**E**) are closed with interruptive sutures in two layers. Clinical appearance of the treated sites at 17-day (**F**), 31-day (**G**) and 45-day (**H**) observation periods.

Anesthesia and surgical procedures were performed as previously described^27^. Anesthesia was induced with 8% isoflurane (Attane™, MINRAD Inc., Orchard Park, NY, USA) and 600 ml/min oxygen in the induction chamber. The rats were placed in the prone position with a non-rebreather facemask (half-open system), maintaining a flow of 3–6% isoflurane with 200 ml/min oxygen. Using an aseptic technique (shaving of the operative area and disinfection with Betadine), local anesthesia with 10% Xylocaine Spray (AstraZeneca, Zug, Switzerland) was administered in the operative field. A mid-sagittal incision was made through the skin and periosteum, and the flaps were carefully reflected from the forehead to expose the calvarial bone (Fig. 9). The distraction devices were placed on the calvarial bones. The periosteum and the skin were sutured over the distraction device in two layers. No device was placed in the sham-operated group. The depth of anaesthesia was determined by observing vital parameters and movement after pain stimulation. A peri-operative dose of 0.1 mg/kg buprenorphin s.c. (Tamgesic®, ESSEX Chemie, Luzern, Switzerland) was continued for four days post-operatively. The animals were monitored daily during the first three weeks and every second day after that until euthanasia on health symptomatology using standardized score sheets.

The distraction device was manipulated under inhalation anesthesia (6% isoflurane and 600 ml/min oxygen). The distraction screw was activated manually by using a hex key. Turning the distraction screw for a quarter of a complete turn (90°) resulted in a 0.1 mm elevation of the plate^27^. For relaxation, the distraction screw was turned back to the same amount as that used for activation. PP protocol consisted of alternated activation with relaxation every 12 h, and the distraction protocol of activation only every 24 h. Animals were subjected to one of the following protocols:

- PDO: distraction (activation every 24 h) during seven days
- DDP group: distraction (activation after 12 h and 24 h) alternated with relaxation (after 36 h) for ten days
- PP_D: PP (alteration of activation and relaxation every 12 h) for three days, followed by distraction (activation at 24 h) for seven days
- D_PP: distraction (activation once in 24 h) for seven days, followed by PP (alteration of activation and relaxation at 12 h) for three days
- PP: PP (alteration of activation and relaxation every 12 h) for ten days
- PE_1: single activation
- Sham: sham-operated animals without device placement

The detailed protocol of device manipulation is presented in Supplementary Table 1.

### Euthanasia

Six rats from each group were euthanized at six observation periods (1, 4, 7, 17, 31, and 45 days after surgery). The animals were euthanized by an overdose of gaseous carbon dioxide in an empty Plexiglas box and observed for at least 10 min after cardiac arrest and breathing cessation. Three animals from each group were used for the real time quantitative polymerase chain reaction (RT-qPCR) analysis. The recovered samples of the other three animals were excised and immediately immersed in a 10% buffered formaldehyde solution with 1% CaCl_2_. The specimens were processed for the µCT and histological analysis.

### RT-qPCR

The skin was elevated from the head, and two samples were collected from each site: the periosteum and bone. The periosteum was incised at the base of the distraction gap and was carefully removed from the distraction device (first sample). The device was then removed. The bone fragment corresponding to the gap region was osteotomized using an oscillating saw and excised from the calvaria. Full bone thickness was used for the analysis (second sample).

Each sample was weighed and stored in an RNA later (Qiagen, Basel, Switzerland). The samples were transferred to a lysis buffer and homogenized three times at 20 Hz for 1 min using a TissueLyser II (Qiagen, Basel, Switzerland). Subsequently, all the samples were placed in cryotubes and stored in liquid nitrogen. Total RNA was prepared using the RNeasy Mini Kit (Qiagen, Basel, Switzerland) according to the manufacturer’s instructions. The RNA was quantified using a NanoDrop 2000 spectrophotometer (Thermo Fisher Scientific). RNA quality was assessed using Bioanalyzer 2100 (Agilent Technologies, Santa Clara, CA, USA). Total RNA was reverse-transcribed using MMLV Reverse Transcriptase (Promega, Dübendorf, Switzerland). Gene expression analysis was performed with quantitative RT-PCR using pre-synthesized TaqMan Gene Expression Assays Sost (Rn00577971_m1), Postn (Rn01494627_m1), b-Ctnnl (Rn01246634_m1), BMP-2 (Rn00567818_m1), ACP5 (Rn00569608_m1), RUNX2 (Rn01512298_m1), SPARC (Rn01470624_m1), collagen I α1 (Rn01463848_m1), and SOX9 (Rn01751070_m1). The expression levels were normalized to those of GAPDH (Rn01775763_g1; Applied Biosystems, Rotkreuz, Switzerland). RT-PCR was performed on a 7500 Fast Real-Time PCR System^#^, and the data were evaluated using sequence detection software SDS (v2.0.1; Life Technologies, Grand Island, NY, USA).

### µCT analysis

**Figure 10:**
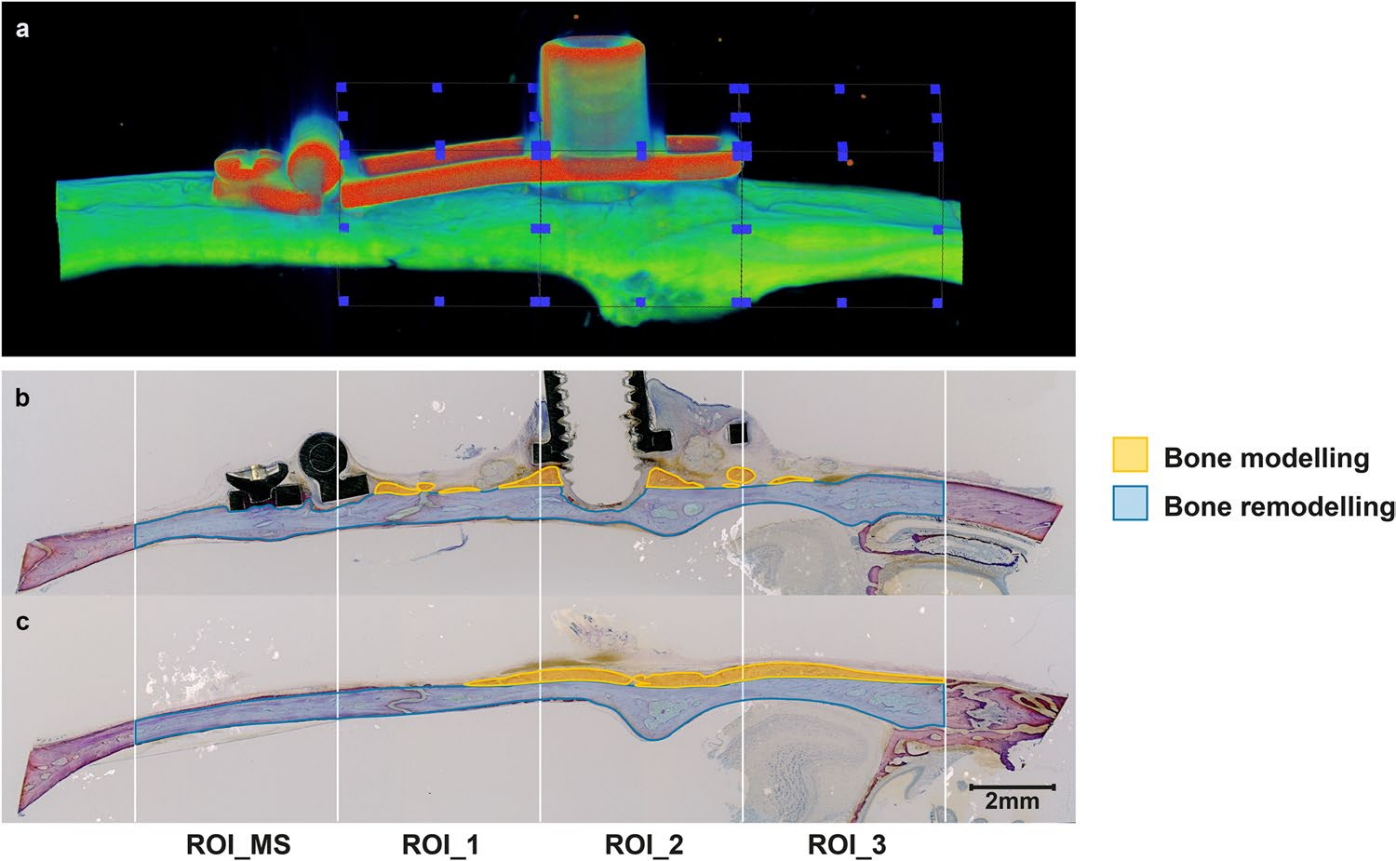
Micro-CT and histomorphometric analysis. (**A**) The volume of interest (VOI) on the micro-CT was measured as a sum of three VOIs: lower part of the distraction gap (VOI_1, 0–5 mm), higher part of the distraction gap (VOI_2, 5–10 mm) and outside the distraction plate (VOI_3). (**B**) The area parameters for the histomorphometric analysis on the central sections for bone modeling (yellow) and bone remodeling (blue) were measured separately and selected manually, using four region of interests (ROIs). The ROI_Sum was measured as a sum of the lower part of the distraction gap (ROI_1, 0–5 mm), higher part of the distraction gap (ROI_2, 5–10 mm) and outside the distraction plate (ROI_3). In addition, a ROI corresponding to of the micro-screw region (ROI_MS) was measured distally to the ROI_1. (**C**) The histological sections outside the distraction plate were projected parallel to the central sections, and the ROIs outlined using the same reference lines.

The distraction sites were subjected to radiography (25 kVP for 10 s) in two projections using a desktop cone-beam scanner (µCT 40, Scanco Medical AG, Brüttisellen, Switzerland). The X-ray source (E) set at 70 kVp with 114 mA at high resolution (1000 projections/180º) showed an image matrix of 2048 × 2048 pixels. The diameter of the sample holder that allowed an increment (resolution) of 15 µm (= voxel size) was 30.7 mm. The integration time was set as 3 s. The µCT slices (1000) were reconstructed perpendicularly to the sagittal axis of the calvarium. Mineralized tissue was selected from grayscale images (0–1000) with a specific threshold of 220 per milles, corresponding to a value of 530 mg HA/cm^3^. Voxels above this value were categorized as mineralized bone, background, or titanium. The entire bone segment underneath the distraction plate was included in the analysis comprising pristine, calvarial bone and newly formed bone, as it was impossible to distinguish old from new bone at the later stages of healing. VOI_Summ was measured as the sum of the three volumes of interest (VOIs) outlined in the lower part of the distraction gap (VOI_1, 0–5 mm), the higher part of the distraction gap (VOI_2, 5–10 mm), and outside the distraction plate (VOI_3, 10–15 mm; Fig. 10a). The reconstructed 2D images were evaluated by 3D segmentation of the VOI, Gaussian Sigma at 0.8, and Gauss support at 1. Cortical contouring was automatically defined using Amira software (Amira, Visualization Sciences Group, Düsseldorf, Germany). For cortical analysis, the following structural parameters were generated: Ct.Th (μm), Ct.BV (mm^3^), Ct.TV (mm^3^), TMD (mg HA/cm^3^), and Ct.Po (%). Ma.V (mm^3^) was calculated as follows: Ct.TV–Ct.BV.

### Histological analysis

The samples were rinsed in running tap water, dehydrated in ascending concentrations of ethanol (40–100%) and xylol and embedded in methylmethacrylate. The embedded tissue blocks were cut along the axis of the distraction device into approximately 800 μm-thick sections using a slow-speed diamond saw (Varicut® VC-50, LECO, St. Joseph, MI, USA). Five tissue slices were prepared from each sample: the most central section throughout the distraction rod, two sections of the distraction plate lateral to the distraction rod, and two sections outside the distraction plate. The sections were mounted onto acrylic glass slabs, grounded, and polished to a final thickness of about 300 μm (Knuth-Rotor-3, Struers, Rodovre/Copenhagen, Denmark). Staining was performed with basic fuchsin and toluidine blue/McNeal. Images were digitally photographed under a light fluorescence microscope connected to a digital imaging system (VHS-6000, Keyence, Japan).

Morphometric analysis of the new and original bone was performed using four ROIs on all sections of each sample, two underneath (ROI_1 and ROI_2) and one outside the distraction plate (ROI_3; Fig. 10b, c). Three ROIs were outlined in relation to the distance from the hinge of the distraction device in the lower part of the distraction gap (ROI_1, 0–5 mm), the higher part of the distraction gap (ROI_2, 5–10 mm), and outside the distraction plate (ROI_3, 10–15 mm;). ROI_Sum was measured as the sum of the three ROIs. In addition, one ROI corresponding to the microscrew (ROI_MS) was measured distal to ROI_1. The area parameters were selected manually and measured separately for bone modeling (new bone apposition) and bone remodeling (within the pristine calvarial bone). The following area parameters of newly formed bone were calculated: WB (including osteoid and woven bone), LB (including parallel-fiberd and lamellar bone), BM (including abundant bone-forming cells), and TNB (WB + LB + BM). The original calvarial bone parameters included newly formed bone (CNB), bone marrow (CBM), old bone (COB), and total bone (TCB). The percentages of the CNB (R_CNB), CBM (R_CBM), and OB (R_COB) relative to the TCB were calculated. The morphometric parameters were distinquished and quantified as previously described^50^. The parameters of µCT and histomorphometry were examined and assessed without being aware of the samples’ allocation.

### Statistical analysis

The Gaussian distribution for each parameter was assessed using the Shapiro–Wilk test. Nonparametric tests were used for non-normally distributed data. The effects of device placement and observation during the latency period on gene expression and their interactions were investigated using a two-way analysis of variance (ANOVA) with subsequent pairwise comparisons. One-way ANOVA with subsequent Tukey's post hoc test was performed to specify the differences in µCT parameters, and the Kruskal–Wallis test with Dunn–Bonferroni's adjustment for bone modeling and remodeling evaluation between the seven groups. The effects of distraction plate elevation (with W/PE [DDP, D_PP, PP_D, and PDO] and without elevation, Wo/PE [PP and PE_1]) and pumping protocol (with W/PP [DDP, D_PP, PP_D, and PP] and without pumping, Wo/PP [PDO and PE_1]) were assessed using two-way ANOVA. Statistical significance was set at α < 0.05. The data are presented as box plots (median and interquartile range). Statistical analyses were performed using SPSS for Windows (Release 19.0, standard version; IBM SPSS, Chicago, IL, USA) and graphed using R software (v. 4.1.2).

### Supplementary Information

Supplementary Information 1.

Supplementary Information 2.

Supplementary Information 3.

Supplementary Information 4.

Supplementary Information 5.

Supplementary Information 6.

Supplementary Information 7.

Supplementary Information 8.

Supplementary Information 9.

## Data Availability

The data that support the findings of this study are available from the corresponding author upon request.
