# Supplementary figures and images for: Alternated activation with relaxation of periosteum stimulates bone modeling and remodeling
Source: Sci Rep. 2024 May 15;14:11136. doi: 10.1038/s41598-024-61902-w (PMC11096315; doi:10.1038/s41598-024-61902-w)

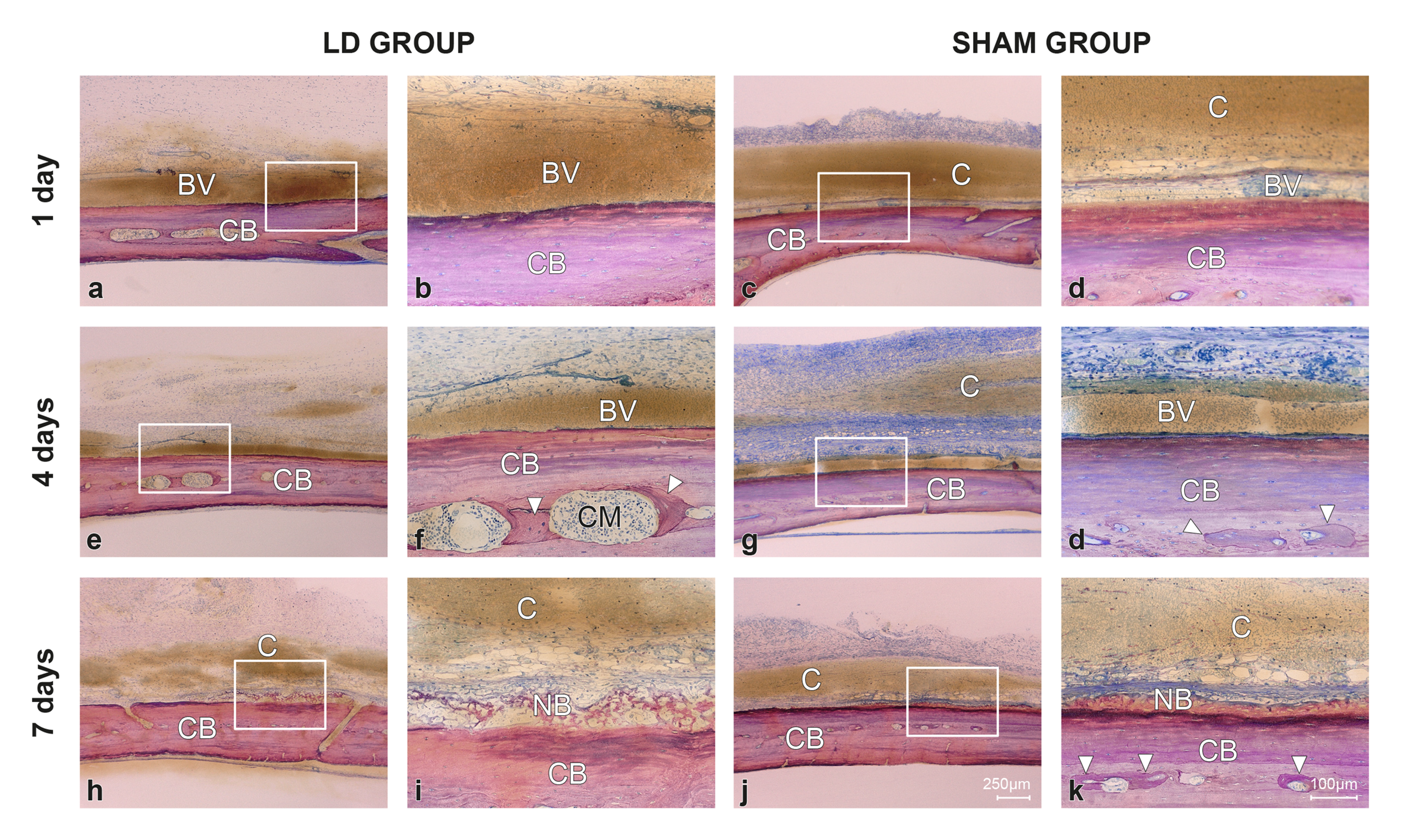

Supplement: Supplementary file 2 — Supplementary Information 2. [file 41598_2024_61902_MOESM2_ESM.tif]

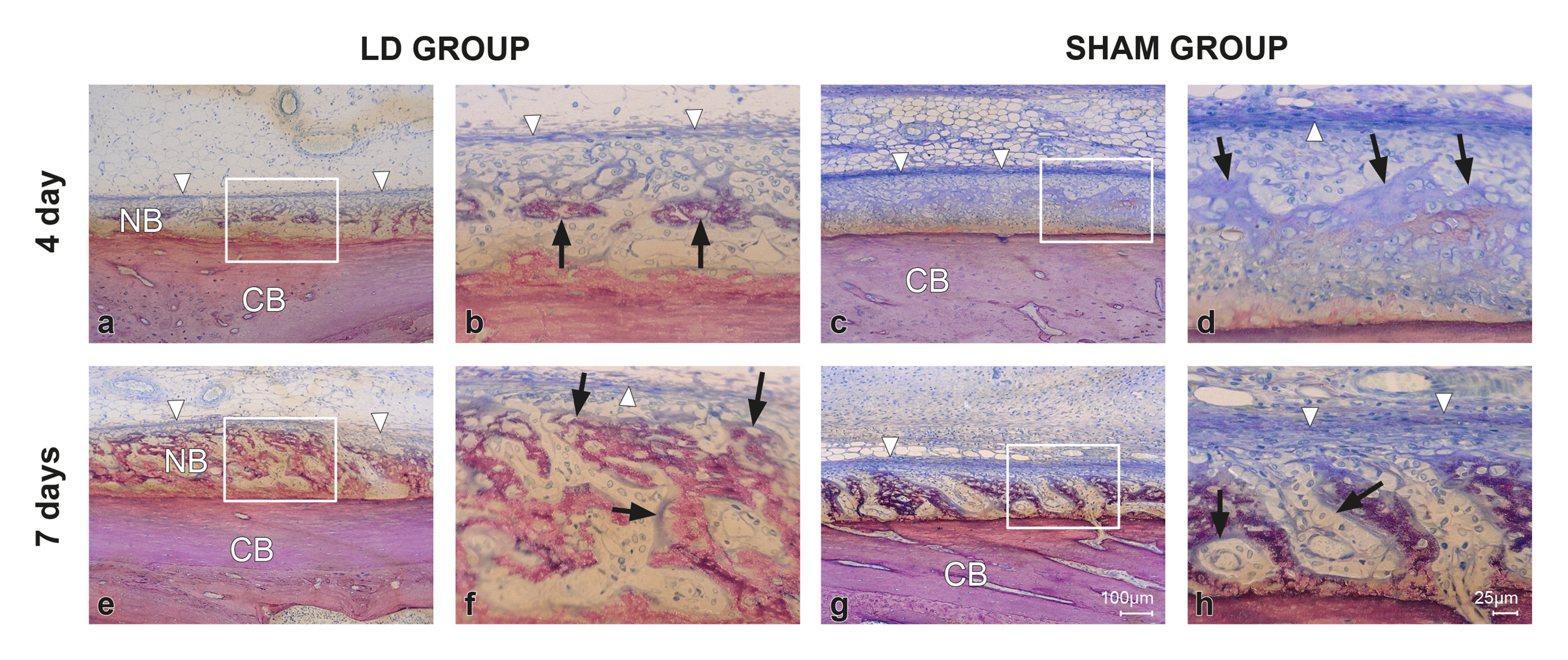

Supplement: Supplementary file 3 — Supplementary Information 3. [file 41598_2024_61902_MOESM3_ESM.tif]

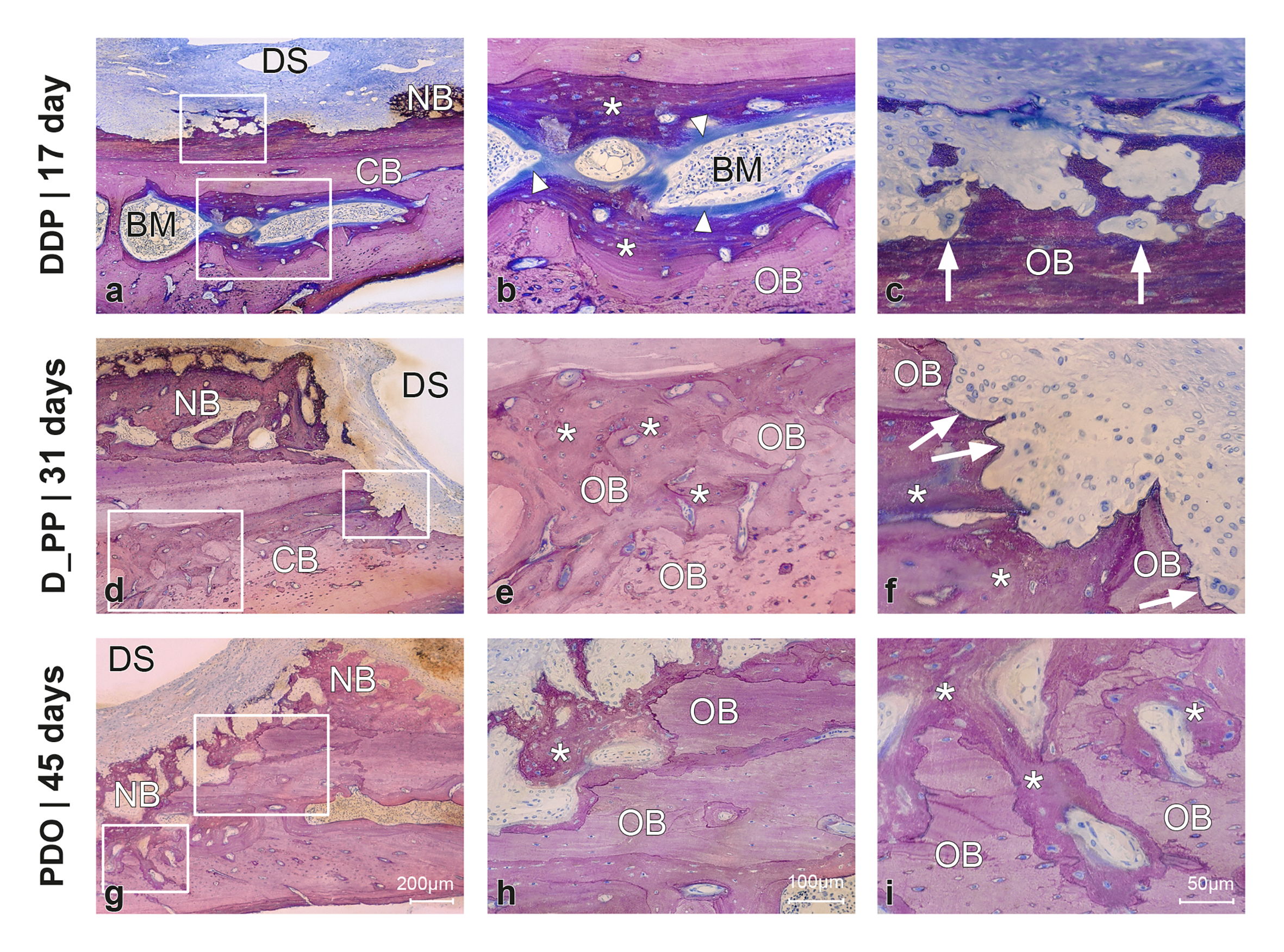

Supplement: Supplementary file 4 — Supplementary Information 4. [file 41598_2024_61902_MOESM4_ESM.tif]

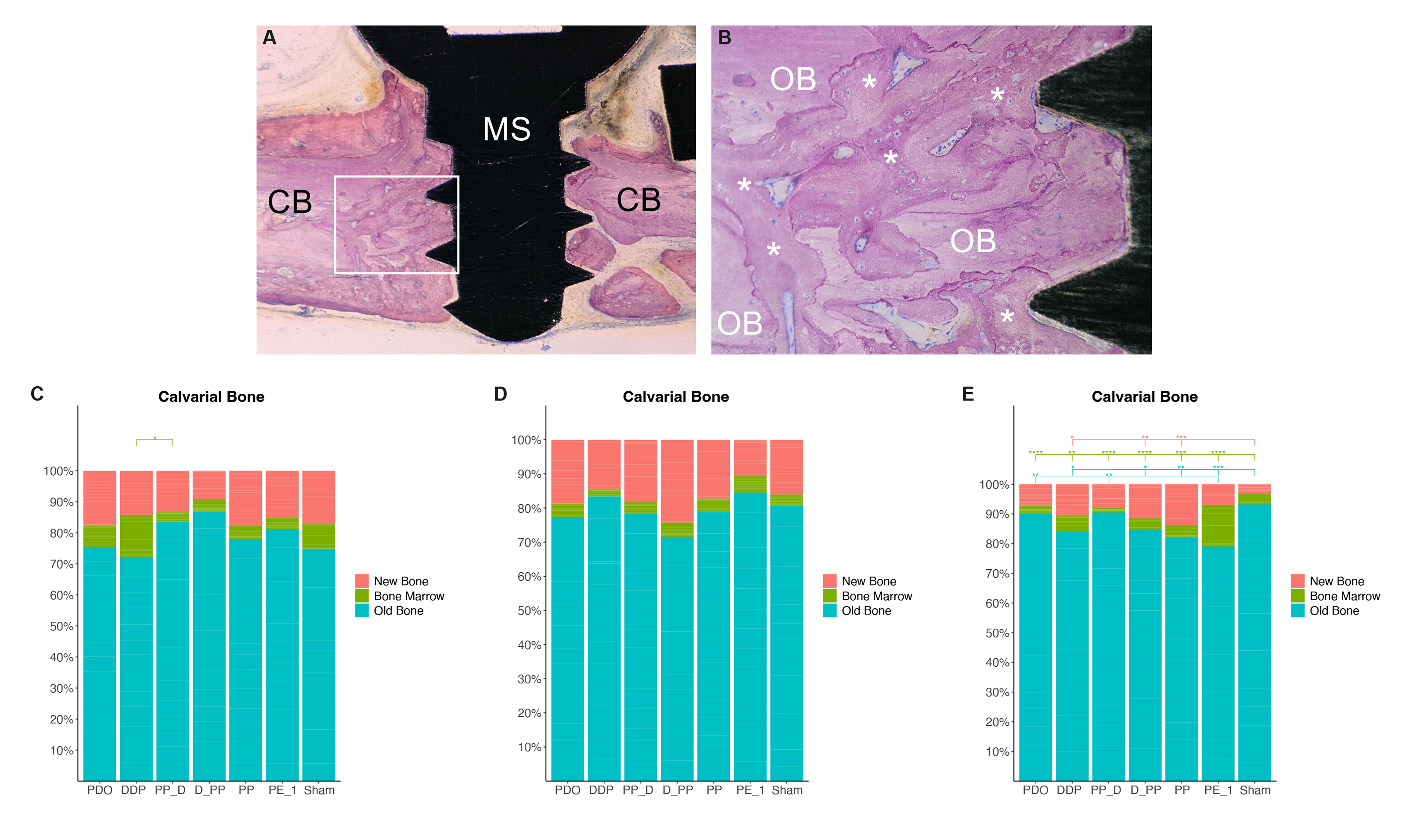

Supplement: Supplementary file 5 — Supplementary Information 5. [file 41598_2024_61902_MOESM5_ESM.tif]
