# Supplementary material for: Alternated activation with relaxation of periosteum stimulates bone modeling and remodeling
Source: Sci Rep. 2024 May 15;14:11136. doi: 10.1038/s41598-024-61902-w (PMC11096315; doi:10.1038/s41598-024-61902-w)
Supplement: Supplementary file 6 — Supplementary Information 6. [file 41598_2024_61902_MOESM6_ESM.docx]

**Supplementary Figure Legend**

**Supplementary Figure S1.** Magnified histological views of the calvarium in the DC and Sham groups at 1-day, 4-day and 7-day observation period (latency period). Longitudinal and transversal view of the blood vessels (BV) running next to the calvarial bone (CB). Remnants of blood clot (C) are visible within the soft tissue at all three observation periods. The boxed areas (left) are magnified (right), showing signs of bone remodelling (arrowheads) at 4-day observation period and minimal apposition of newly formed bone (NB) at 7-day observation period. Toluidine blue and fuchsin staining.

**Supplementary Figure S2.** Magnified histological images of newly formed bone (NB) in the LD and Sham groups at 4-day and 7-day observation period (latency period). The boxed areas (left) are magnified (right), illustrating initial bone formation with osteoid and osteoblasts (arrows) between the periosteum (arrowheads) and calvarial bone (CB). Toluidine blue and fuchsin staining.

**Supplementary Figure S3.** Magnified histological images of the sites where rod of the distraction screw (DS) was in contact with the calvarial bone (CB) in ROI_2 at 17-day, 35-day and 41-day observation period. Formation of new bone (NB) occurred next to the resorption site. The boxed areas (A, D, G) are magnified (B, E, H and C, F, I, respectively). New bone (*) within the old bone (OB) is formed in association with the resorption processes observed lateral to the distraction screw. Osteoid (arrowheads) is clearly visible at the leading front of bone formation, lining the bone marrow (BM) cavity. Numerous Howship’s lacunae and osteoclasts are present at the resorption sites (arrows). Toluidine blue and fuchsin staining.

**Supplementary Figure S4.** The remodeling-based bone formation in the ROI_MS corresponding to the micro-screw region. Micro-screw (MS) is integrated in the calvarial bone (CB). New bone (*) within the old bone (OB) is formed next to the micro-screw. No statistically significant difference between the parameters was detected at the 17-day and 31-day observation periods. At the 45-day observation period, the highest R_CNB were detected in the DDP, D_PP and PP groups, while R_CBM in the PE_1 group and R_OCB in the Sham group.
