# Supplementary material for: Alternated activation with relaxation of periosteum stimulates bone modeling and remodeling
Source: Sci Rep. 2024 May 15;14:11136. doi: 10.1038/s41598-024-61902-w (PMC11096315; doi:10.1038/s41598-024-61902-w)
Supplement: Supplementary file 7 — Supplementary Information 7. [file 41598_2024_61902_MOESM7_ESM.docx]

| **Supplementary Table 1. Protocol of periosteal manipulation** | | | | | | | | | | | | | | | | | | | | | |  |
| --- | --- | --- | --- | --- | --- | --- | --- | --- | --- | --- | --- | --- | --- | --- | --- | --- | --- | --- | --- | --- | --- | --- |
| Group | Day: | 1 | | 2 | | 3 | | 4 | | 5 | | 6 | | 7 | | 8 | | 9 | | 10 | | Gap size (mm) |
|  | Hour: | 7h | 19h | 7h | 19h | 7h | 19h | 7h | 19h | 7h | 19h | 7h | 19h | 7h | 19h | 7h | 19h | 7h | 19h | 7h | 19h |  |
| PDO |  | A | - | A | - | A | - | A | - | A | - | A | - | A | - | - | - | - | - | - | - | 0.7 |
| DDP |  | A | A | R | A | A | R | A | A | R | A | A | R | A | A | R | A | A | R | A | A | 0.7 |
| PP_D |  | A | R | A | R | A | R | A | - | A | - | A | - | A | - | A | - | A | - | A | - | 0.7 |
| D_PP |  | A | - | A | - | A | - | A | - | A | - | A | - | A | - | A | R | A | R | A | R | 0.7 |
| PP |  | A | R | A | R | A | R | A | R | A | R | A | R | A | R | A | R | A | R | A | R | 0 |
| PE_1 |  | A | - | - | - | - | - | - | - | - | - | - | - | - | - | - | - | - | - | - | - | 0.1 |
| Sham |  | - | - | - | - | - | - | - | - | - | - | - | - | - | - | - | - | - | - | - | - | - |
| A: activation at 0.1 mm; R: relaxation at 0.1 mm. | | | | | | | | | | | | | | | | | | | | | |  |
