# Supplementary material for: Alternated activation with relaxation of periosteum stimulates bone modeling and remodeling
Source: Sci Rep. 2024 May 15;14:11136. doi: 10.1038/s41598-024-61902-w (PMC11096315; doi:10.1038/s41598-024-61902-w)
Supplement: Supplementary file 8 — Supplementary Information 8. [file 41598_2024_61902_MOESM8_ESM.docx]

| **Healing Period** | **Parameter** | **VOI_1** | **VOI_2** | **VOI_3** | **VOI_Summ** |
| --- | --- | --- | --- | --- | --- |
| 17-day | Cr.Th | .052 | **.002** | .104 | **.003** |
|  | Cr.BV | .052 | **.007** | .093 | **.008** |
|  | Cr.TV | **.029** | **.007** | .100 | **.004** |
|  | TMD | **.027** | **.037** | .478 | **.017** |
|  | Cr.Po | .117 | .587 | .553 | .052 |
|  | Ma.V | .479 | .078 | .549 | .143 |
| 31-day | Cr.Th | .391 | **<.001** | .192 | **.008** |
|  | Cr.BV | .391 | **<.001** | .404 | **.031** |
|  | Cr.TV | .219 | **<.001** | .466 | **.020** |
|  | TMD | .789 | .071 | .054 | .805 |
|  | Cr-Po | .471 | .087 | .170 | .193 |
|  | Ma.V | **<.001** | **.005** | .663 | **.043** |
| 45-day | Cr.Th | .266 | **.008** | .378 | .056 |
|  | Cr.BV | .266 | **.010** | .156 | .067 |
|  | Cr.TV | .095 | **.004** | .165 | **.024** |
|  | TMD | **<.001** | .424 | .061 | .051 |
|  | Cr-Po | **.001** | .339 | **.015** | **.010** |
|  | Ma.V | .061 | **.003** | .168 | **.028** |

**Supplementary Table S2.** One-way ANOVA Univariate t – test for Three Healing Periods After Division of the Volume of Interest Into Three Subvolumes.

One-way ANOVA p-values for the micro-CT parameters. Means ± SD are shown. Three volume of interests (VOIs) were outlined into lower part of the distraction gap (VOI_1, 0 - 5 mm), higher part of the distraction gap (VOI_2, 5 – 10 mm) and outside the distraction plate (VOI_3, 10 – 15 mm). VOI_Summ = VOI_1 + VOI_2 + VOI_3. Ct.Th = cortical thickness; Ct.BV = cortical bone volume, Ct.TV = cortical tissue volume, TMD = tissue mineral density; Ct.Po = cortical porosity; Ma.V = Ct.TV – Ct.BV = marrow volume.

.
