# Supplementary material for: Alternated activation with relaxation of periosteum stimulates bone modeling and remodeling
Source: Sci Rep. 2024 May 15;14:11136. doi: 10.1038/s41598-024-61902-w (PMC11096315; doi:10.1038/s41598-024-61902-w)
Supplement: Supplementary file 9 — Supplementary Information 9. [file 41598_2024_61902_MOESM9_ESM.docx]

| **Healing Period** | **Parameter** | **ROI_1** | **ROI_2** | **ROI_3** | **ROI_Summ** |
| --- | --- | --- | --- | --- | --- |
| 17-day | WB | .075 | **<.001** | **<.001** | **<.001** |
|  | LB | .407 | .124 | .749 | .390 |
|  | BM | .755 | **<.001** | **<.001** | **<.001** |
|  | TNB | .443 | **<.001** | **<.001** | **<.001** |
|  | R_CNB | **.001** | .105 | .145 | **.016** |
|  | R_CBM | .198 | **.002** | .007 | **<.001** |
|  | R_COB | .004 | <.001 | .003 | **<.001** |
| 31-day | WB | **<.001** | **<.001** | **.009** | **<.001** |
|  | LB | **<.001** | **.001** | **.024** | **<.001** |
|  | BM | **<.001** | **<.001** | .111 | **<.001** |
|  | TNB | **<.001** | **<.001** | **.001** | **<.001** |
|  | R_CNB | .787 | **.001** | **.045** | **.016** |
|  | R_CBM | .690 | .105 | .088 | **.032** |
|  | R_COB | .768 | **<.001** | .453 | **.018** |
| 45-day | WB | **.005** | **<.001** | **.025** | **<.001** |
|  | LB | **<.001** | **<.001** | **<.001** | **<.001** |
|  | BM | **<.001** | **<.001** | **.036** | **<.001** |
|  | TNB | **<.001** | **<.001** | **<.001** | **<.001** |
|  | R_CNB | **.018** | **<.001** | **.013** | **<.001** |
|  | R_CBM | **<.001** | **.003** | .103 | .165 |
|  | R_COB | **.004** | **<.001** | .110 | **.008** |

**Supplementary Table S3.** Kruskal-Wallis Test for Three Healing Periods After Division of the Region of Interest Into Three Subregions.

Kruskal-Wallis Test *p*-values for the morphometric parameters. Means ± SD are shown. Three region of interests (ROIs) were outlined into lower part of the distraction gap (ROI_1, 0 - 5 mm), higher part of the distraction gap (ROI_2, 5 – 10 mm) and outside the distraction plate (ROI_3, 10 – 15 mm). ROI_Summ = ROI_1 + ROI_2 + ROI_3. WB = new woven bone; LB = new lamellar bone; BM = new bone marrow; TNB = total new bone; R_CNB = relative % of calvarial new bone to total calvarial bone; R_CBM = relative % of calvaril bone marrow to total calvarial bone; R_COB = relative % of old calvarial bone to total calvarial bone.

17- day observation period

The Sham group showed the lowest values for WB, BM and TNB in ROI_2 being highly significant to all except for the PP group (p = 0.021), which showed lower WB (p = 0.036) and TNB (p = 0.038) than the PDP group. The highest differences in ROI_3 were observed for WB, BM and TNB between the PP and Sham groups, being higher for the PP group (p = 0.001).

Highest differences were observed between the Sham group showing lower R_OCB and higher R_NCB compared to the D_PP group in ROI_1 (p = 0.015 and p = 0.008, respectively), lower R_OCB and higher R_CBM compared to PE_1 in ROI_2 (p = 0.001 and p = 0.002, respectively) and compared to the D_PP group in ROI_3 (p = 0.004 and p = 0.011, respectively).

No significant differences were observed in ROI_MS (Supplemental Figure 6).

31- day observation period

D_PP group reached highly significant difference compared to the Sham group for WB, BM and TNB in ROI_1 (p < 0.001) and ROI_2 (p < 0.001), and for WB (p = 0.004) and TNB (p < 0.001) in ROI_3. Furthermore, the D_PP showed higher WB in ROI_1 (p = 0.006) and in ROI_2 (p = 0.018), and more BM (p = 0.002) and TNB (p = 0.012) in ROI_2 than the PE_1 group.

Compared to PDO and PP_D groups, D_PP group showed lower R_OCB (p = 0.003 and p = 0.001, respectively) and higher R_NCB (p = 0.003 and p = 0.001, respectively) in ROI_2.

D_PP group showed more R_NCB than the PE_1 in the ROI_MS (Supplemental Figure 6).

45-day observation period

PDO and D_PP groups reached the high statistical significance for TNB compared to the Sham in all three ROIs. PE_1 group showed significantly lower LB than the PDP (p = 0.015) and TNB than the PDO group (p = 0.049) in ROI_1, and lower LB than PP_D (p = 0.041) and PDP (p = 0.002) groups in ROI_2.

The R_CNB was highest in the PP group and lowest in the Sham group in all three ROIs. The PP_D group demonstrated more R_CBM in ROI_1 (p = 0.003) and ROI_2 (p = 0.003), and the PP group more R_NCB in ROI_1 (p = 0.011), ROI_2 (p = 0.001) and ROI_3 (p = 0.048) as compared to the Sham group.

In the ROI_MS, the PP_D, D_PP and PP groups showed similar R_CNB values, being significantly different in comparison to the Sham group (Supplemental Figure 6).

*W/PE vs. Wo/PE*

W/PE reached more WB (p = 0.036) and TNB (p = 0.037) ROI_2 than the Wo/PE group at 17-day post-surgery. Furthermore, W/PE group showed less R_OCB (p = 0.048) in ROI_2 than the Wo/PE group.

All parameters were higher for W/PE than Wo/PE group at 31-day post-surgery, reaching the significance for BM in ROI_2 (p = 0.013), for WB in ROI_1 (p = 0.035) and ROI_2 (P = 0.005) and TNB in ROI_2 (p = 0.023).

The W/PE group reached significantly higher values than the Wo/PE group for LB in ROI_1 (p = 0.003) and ROI_2 (P = 0.008), BM in ROI_2 (P = 0.040) and TNB in ROI_1 (P = 0.002) and ROI_2 (p = 0.0028) at 45-day post-surgery. The R_NCB was significantly higher in Wo/PE than the W/PE group in ROI_2 (p = 0.001) and ROI_3 (p < 0.001).

*W/PP vs. Wo/PP*

The W/PP group showed more BM in ROI_1 (p = 0.027) and ROI_2 (p = 0.046), and WB in in ROI_1 (p = 0.025) and LB (p = 0.008) than the Wo/PP group at 31-day post-surgery.

The R_NCB was significantly higher in W/PP than in Wo/PP group in ROI_1 (p = 0.033) at 45-day post-surgery.
